# Effective Singlet Oxygen Sensitizers Based on the Phenazine Skeleton as Efficient Light Absorbers in Dye Photoinitiating Systems for Radical Polymerization of Acrylates

**DOI:** 10.3390/ma14113085

**Published:** 2021-06-04

**Authors:** Ilona Pyszka, Zdzisław Kucybała, Beata Jędrzejewska

**Affiliations:** Faculty of Chemical Technology and Engineering, UTP University of Science and Technology, 85-326 Bydgoszcz, Poland; kucybala@utp.edu.pl

**Keywords:** phenazine derivatives, sensitizers, singlet oxygen, dye photoinitiating systems, radical polymerization

## Abstract

A series of dyes based on the phenazine skeleton were synthesized. They differed in the number of conjugated double bonds, the arrangement of aromatic rings (linear and/or angular system), as well as the number and position of nitrogen atoms in the molecule. These compounds were investigated as potential singlet oxygen sensitizers and visible light absorbers in dye photoinitiating systems for radical polymerization. The quantum yield of the singlet oxygen formation was determined by the comparative method based on the ^1^H NMR spectra recorded for the tested dyes in the presence of 2,3-diphenyl-*p*-dioxene before and after irradiation. The quantum yield of the triplet state formation was estimated based on the transient absorption spectra recorded using the nanosecond flash photolysis technique. The effectiveness of the dye photoinitiating system was characterized by the initial rate of trimethylolpropane triacrylate (TMPTA) polymerization. In the investigated photoinitiating systems, the sensitizer was an electron acceptor, whereas the co-initiator was an electron donor. The effectiveness of TMPTA photoinitiated polymerization clearly depended on the arrangement of aromatic rings and the number of nitrogen atoms in the modified phenazine structure as well as the quantum yield of the triplet state formation of the photosensitizer in the visible light region.

## 1. Introduction

Oxygen is the most abundant element on Earth and is involved in many chemical and physical processes. Alchemists called it the “food of life” since it is necessary for life. Despite its huge positive role, oxygen can also destroy cell structures, which, in turn, contributes to the aging of organisms and the development of cancer. Changes in the properties of oxygen and its ability to initiate a number of processes are conditioned by the presence of light. Because of the interaction of electromagnetic radiation with oxygen, reactive oxygen species (ROS), widely described in literature, may be formed [1]. They are highly energetic, and the excess energy is responsible for their high reactivity. One such form is singlet oxygen.

In the ground state, the oxygen molecule has two unpaired electrons having their spins parallel and occupying perpendicular anti-bonding orbitals π, so it is in the triplet form (^3^O_2_). Thus, the O_2_ molecule is paramagnetic—the spin quantum number is 1. When the molecule gains energy upon excitation, the electron spins are paired, and the electron configuration of the oxygen molecule changes from a triplet to a singlet ^1^O_2_. Thus, in the excited state, the spin quantum number is 0. Such a system of energy levels distinguishes the oxygen molecule from the vast majority of organic compounds for which the electronic ground state is the singlet state S_0_, whereas the lowest excited energy state is the triplet state T_1_ [2,3,4].

This chemically useful singlet oxygen form can be prepared by two main methods, i.e., irradiation of the ^3^O_2_ form and chemical synthesis. The latter method produces the singlet oxygen as one of the products of a chemical reaction. The following examples can be given: (i) the reaction of hydrogen peroxide with sodium chlorate (I) [5,6,7], (ii) the decomposition of some peroxides [8,9,10], and (iii) the decomposition of adducts of organophosphorus compounds and ozone [11,12]. The other way is to produce singlet oxygen by an atmospheric discharge [13,14]. In nature, singlet oxygen is commonly formed from water during photosynthesis, using the energy of sunlight [15]. It is also produced in the troposphere by the photolysis of ozone by light of short wavelength [16] and by the immune system as a source of active oxygen [17]. 

However, a more interesting way to generate singlet oxygen is a photochemical process based on an excitation of triplet oxygen with ultraviolet light in the presence of a sensitizing dye. In that case, oxygen molecule takes energy from the sensitizer being in the triplet excited state. A singlet oxygen sensitizer can be any compound that forms a triplet state and fulfils the following conditions:

1. It does not react with oxygen, i.e., it does not undergo auto-oxidation [18,19];
2. It has a high molar absorption coefficient at the excitation wavelength and the appropriate spectral characteristics enabling selective excitation of the sensitizer, while limiting the excitation of other molecules present in the solution;
3. It has a low triplet state energy [20].

The above conditions are met by a number of organic compounds that are effective singlet oxygen sensitizers, e.g., erythrosine, methylene blue, chlorophyll, hematoporphyrin, rose bengal, perinaftenone, acridine, and others [21,22,23].

Singlet oxygen sensitizers usually operate in the same phase in which the photooxidation is performed. In that case, the sensitization reaction is carried out in solution. However, a number of heterogeneous sensitizers have now been developed. Their use allows for photooxidation without side effects. There are several ways to prepare heterogeneous sensitizers. One of them is using a polymeric carrier (e.g., chloromethylated polystyrene beads or modified silica gel) to which the sensitizer is chemically attached [24,25].

Upon excitation the sensitizer molecule undergoes a change from the singlet ground state S(S_0_) to the singlet excited state *S(S_1_).

S(S_0_) + hν → *S(S_1_) (1)

As a result of the energy transfer (or electron transfer) from the sensitizer molecule in its excited singlet state *S(S_1_) to the ground-state oxygen molecule ^3^O_2_ through collisions, the sensitizer molecule goes to the triplet state *S(T_1_). The singlet oxygen formed in the excited state can oxidize other compounds.

*S(S_1_) + ^3^O_2_ → *S(T_1_) + ^1^O_2_ (^1^Δg) (2)

Only those sensitizers with a lifetime of the S_1_ excited state greater than ns will be effective in interacting with oxygen molecules. Furthermore, when the energy gap for the sensitizer is lower than the triplet-singlet energy difference for the oxygen molecule, the process described by Equation (2) is not allowed energetically. In such a case, the oxygen molecule may be a medium participating in the formation of the triplet state of the sensitizer molecule (Equation (3)) or may influence the process of deactivating the excited singlet state of the sensitizer *S(S_1_) to the ground state S(S_0_) (Equation (4)). Both of these processes can occur through the formation of an exciplex. Polycyclic aromatic hydrocarbons mostly undergo the processes described by Equations (3) and (4) because of the insufficient energy difference between the states S_1_ and T_1_.

*S(S_1_) + ^3^O_2_ → *S(T_1_) + ^3^O_2_ (3)

*S(S_1_) + ^3^O_2_ → S(S_0_) + ^3^O_2_ (4)

The most effective method of generating singlet oxygen under laboratory conditions is the triplet (T_1_) quenching of the sensitizer molecule. This method plays an important role in many applications of oxygen.

The study of reactive oxygen species is crucial for several groups of scientists, including photochemists, biologists, and physiologists [2,26,27,28,29,30]. The search for new, efficient singlet oxygen sensitizers seems to be important because of the positive and negative role of singlet oxygen for humans. Singlet oxygen is used in medicine because it can be involved, for example, in the destruction of cancer cells [23]. In addition, photo-oxidation occurring under the influence of singlet oxygen is used to sterilize blood and surgical instruments as well as to purify water [22,31]. The negative effects of singlet oxygen include polymer degradation and photo-oxidation of food products [32].

In the present work, our intention was to apply dyes based on the phenazine skeleton, which effectively generated singlet oxygen, as visible light absorbers in the photoinitiating systems for radical polymerization. Such dye photoinitiators operate through a long-lived triplet state, which is beneficial in terms of generating radicals through the electron transfer process. Therefore, we performed a comparative study on the quantum yield of the triplet state formation of the phenazine derivatives. We investigated the dye-sensitized photooxidation of 2,3-diphenyl-1,4-dioxene (DPD) to determine the quantum yield of singlet oxygen as it is equal to the quantum yield of triplet state formation when large excess oxygen is used. Additionally, we used a nanosecond flash photolysis technique since the analysis of transient absorption spectra allowed us to determine the quantum yield of the triplet state formation as well.

We have attempted to test phenazine derivatives as photosensitizers in radical polymerization, as new dye structures are still being sought because of the high demands placed on photoinitiators in modern applications such as dental filling materials, reprography (photoresists, printing plates, integrated circuits), laser-induced 3D curing, holographic recordings, and nanoscale micromechanics. Modifications of the photosensitizer structure were carried out by introducing heavy atoms and electron-donating or electron-withdrawing substituents to various heterocyclic rings [33,34,35,36,37,38,39,40,41,42,43] or by the preparation of new chemical skeletons. Our strategy for synthesis followed the modification of phenazine structure by increasing either nitrogen atoms to 3 or 4 in the dye molecule or aromatic rings in the angular and/or linear positions. Such phenazine-functionalized dyes fulfilled the requirements for photoinitiators of radical polymerization, i.e., they exhibited high molar absorption coefficients in visible light, efficiently interacted with additives (e.g., electron donors), and revealed a long-lived triplet state. Moreover, their activity in the visible light region allowed them to meet economic and environmental constraints because visible light is safer, cheaper, and more easily available than ultraviolet light; therefore, inexpensive irradiation setups like low-power light-emitting diodes (LEDs) and diode-pumped solid-state (DPSS) lasers can be used to initiate the polymerization reaction [44].

The synthesized dyes were used as a primary light absorber, which formed the photoinitiating system with a suitable co-initiator. In our study, we used commercially available [(3,4-dimethoxyphenyl)sulfanyl]acetic acid as the co-initiator.

## 2. Materials and Methods

### 2.1. Reagents

Dye sensitizers based on the phenazine skeleton (FN1–FN13) (see Table 1) were synthesized by the condensation of appropriate diamines with quinones using the method described in the literature [45,46,47,48,49,50,51,52,53,54,55]. The crude product was purified by recrystallization from ethyl acetate. Reagents for synthesis: 1,2-diaminobenzene and 2,3-diaminonaphthalene were purchased from Alfa Aesar Co., Ward Hill, MA, United States; 9,10-phenanthrenequinone, 1,2-naphthoquinone, 9,10-diaminophenanthrene, 2,3-diaminopyridine, 4, 5-diamino-pyrimidine, 4-bromo-1,2-diaminobenzene, and 2,3-diamino-5-bromopyridine were purchased from Sigma-Aldrich Co, Saint Louis, MO, United States; 1,2-diammonocyclohexane and 1,10-phenanthroline-5,6-dione were purchased from Acros Organics Co, Pittsburgh, PA, United States; and 5-bromo-2,3-diaminopyrazine was purchased from Fluorochem Co, Hadfield, UK.

All solvents for synthesis, photochemical studies, and NMR analysis, including acetic acid, ethyl acetate, deuterated chloroform (CDCl_3_), chloroform, and 1-methyl-2-pyrrolidinone (MP), were purchased from Sigma-Aldrich Co, Saint Louis, MO, United States. The DC-Plastikfolien Silica gel 60 F254 thin layer chromatography plates (0.2 mm) were from Merck Co, Kenilworth, NJ, USA.

Camphorquinone (CQ, initiator), [(3,4-dimethoxyphenyl)sulfanyl]acetic acid (MKTFO, co-initiator), and trimethylolpropane triacrylate (TMPTA, monomer) were obtained from Sigma-Aldrich Co, Saint Louis, MO, USA.

### 2.2. Synthesis

*10,11,12,13-Tetrahydrodibenzo[a,c]phenazine-FN1*

1,2-diaminocyclohexane (0.548 g, 4.8 mmol) was refluxed with 9,10-phenrenequinone (1 g, 4.8 mmol) in glacial acetic acid (40 mL) for 2 h. The compound was obtained as yellow crystals: C_20_H_18_N_2_, 286.38 g/mol, 1.12 g, yield 82%, m.p. 174–176 °C, lit. 173 °C [51].

^1^H NMR (400 MHz, CDCl_3_, δ): 9.24–9.21 (d, 1H), 8.62–8.59 (d, 2H), 7.78–7.70 (m, 4H), 3.28–3.25 (m, 5H), 2.02–2.08 (m, 5H)

^13^C NMR (100 MHz, CDCl_3_, δ): 152.1, 138.2, 135.8, 131.0, 129.2, 129.1, 127.5, 125.23, 122.6, 32.4, 22.75

*Dibenzo[a,c]phenazine-FN2*

1,2-diaminobenzene (0.519 g, 4.8 mmol) was refluxed with 9,10-phenrenequinone (1 g, 4.8 mmol) in glacial acetic acid (80 mL) for 2 h. The compound was obtained as pale, yellow crystals: C_20_H_12_N_2_, 280.33 g/mol, 1.34 g, yield 80%, m.p. 224–225 °C, lit. 224–226 °C [56].

^1^H NMR (400 MHz, CDCl_3_, δ): 9.34 (d, 2H), 9,32 (d, 2H), 8.50–8.48 (d, 2H), 8.27–8.24 (m, 2H), 7.870–7.65 (m, 6H)

^13^C NMR (100 MHz, CDCl_3_, δ): 141.8, 141.3, 132.0, 130.7, 130.2, 129.3, 128.9, 128.0, 126.5, 122,9

*Tribenzo[a,c,i]phenazine-FN3*

2,3-diaminonaphthalene (0.76 g, 4.8 mmol) was refluxed with 9,10-phenrenequinone (1 g, 4.8 mmol) in glacial acetic acid (100 mL) for 2 h. The compound was obtained as yellow crystals: C_24_H_14_N_2_, 330.39 g/mol, 1.36 g, yield 86%, m.p. 292–293 °C, lit. 291–293 °C [57].

^1^H NMR (400 MHz, CDCl_3_, δ): 9.38–9.36 (d, 2H), 8.88 (s, 2H), 8,47–8.45 (d, 2H), 8.14–8.11 (m, 4H), 7.76–7.51 (m, 4H)

*Tribenzo[a,c,h]phenazine-FN4*

1,2-naphthoquinone (0.76 g, 4.8 mmol) was refluxed with 9,10-phenrenequinone (1 g, 4.8 mmol) in glacial acetic acid (150 mL) for 1 h. The compound was obtained as yellow crystals: C_24_H_14_N_2_, 330.39 g/mol, 1.0 g, yield 63%, m.p. 257–258 °C, lit. 278–279 °C [58].

^1^H NMR (400 MHz, CDCl_3_, δ): 9.78 (d, 1H), 9.62 (d, 2H), 8.93 (d, 1H), 8.69 (m, 2H), 8.47-8.44 (m, 2H), 8.23 (d, 2H), 7.93-7.86 (m, 4H)

*Tetrabenzo[a,c,h,i]phenazine-FN5*

9,10-diaminophenanthrene (1.0 g, 4.8 mmol) was refluxed with 9,10-phenrenequinone (1 g, 4.8 mmol) in glacial acetic acid (150 mL) for 2 h. The compound was obtained as yellow crystals: C_28_H_16_N_2_, 380.45 g/mol, 1.1 g, yield 60%, m.p., lit. 462 °C [59].

^1^HNMR (400 MHz, DMSO-*d*_6_, δ): 8.33–8.31 (d, 4H), 8.05 (d, 2H), 8.03 (d, 2H), 7.82–7.77 (d, 5H), 7.56–7.53 (m, 3H)

*Benzo[a]phenazine-FN6*

1,2-diaminobenzene (0.68 g, 6.3 mmol) was refluxed with 1,2-naphthoquinone (1 g, 6.3 mmol) in glacial acetic acid (100 mL) for 2 h. The compound was obtained as yellow crystals: C_16_H_10_N_2_, 230.27 g/mol, 0.94 g, yield 65%, m.p. 230–232°C, lit. 230–233 °C [52].

^1^HNMR (400 MHz, CDCl_3_, δ): 9.65 (1H, d), 8.50 (1H, d), 8.37 (1H, d), 8.23 (1H, d), 7.99–7.60 (m, 6H)

^13^C NMR (100 MHz, CDCl_3_, δ): 142.9, 142.5, 132.9, 131.1, 131.0, 130.6, 130.3, 129.9, 128.6, 128.2, 127.4, 125.80 

*Dibenzo[a,i]phenazine-FN7*

2,3-diaminonaphthalene (1.0 g, 6.3 mmol) was refluxed with 1,2-naphthoquinone (1 g, 6.3 mmol) in glacial acetic acid (100 mL) for 2 h. The compound was obtained as yellow crystals: C_20_H_12_N_2_, 280.33 g/mol, 0.94 g, yield 87%, m.p. 247–248 °C, lit. 247 °C [60].

^1^HNMR (400 MHz, CDCl_3_, δ): 9.32 (d, 1H), 9.03 (s, 1H), 8.94 (s, 1H), 8.15 (m, 3H), 8.03 (d, 1H), 7.86–7.77 (m, 3H), 7.61–7.59 (m, 2H)

^13^C NMR (100 MHz, CDCl_3_, δ): 143.9, 138.7, 136.8, 134.7, 134.2, 132.8, 130.8, 130.5, 129.0, 128.8, 128.5, 128.4, 128.1, 127.7, 127.2, 125.6, 124.6, 124.4

*Dibenzo[f, h]pyrido[2, 3-b]quinoxaline-FN8*

1,2-diaminopyridine (0.53 g, 4.8 mmol) was refluxed with 9,10-phenanthrenequinone (1 g, 4.8 mmol) in glacial acetic acid (80 mL) for 2 h. The compound was obtained as yellow crystals: C_19_H_11_N_3_, 281.32 g/mol, 1.1 g, yield 81%, m.p. 218–219 °C, lit. 217–219 °C [48,49].

^1^HNMR (400 MHz, CDCl_3_, δ): 9.52-9.49 (d, 1H), 9.31-9.27 (m, 2H), 8.66-8.64 (d, 1H), 8.52-8.50 (d, 2H), 7.84-7.68 (m, 5H)

^13^C NMR (100 MHz, CDCl_3_, δ): 154.4, 149.8, 145.0, 143.6, 138.3, 137.3, 132.5, 132.3, 131.3, 130.8, 129.7, 129.5, 128.1, 128.0, 127.3, 126.4, 124.8, 123.0, 122.8

*11-Bromodibenzo[a, c]phenazine-FN9*

4-bromo-1,2-diaminobenzene (0.898 g, 4.8 mmol) was refluxed with 9,10-phenanthrenequinone (1 g, 4.8 mmol) in glacial acetic acid (80 mL) for 30 min. The compound was obtained as yellow crystals: C_20_H_11_BrN_2_, 359.23 g/mol, 1.24 g, yield 72%, m.p. 274–275 °C, lit. 274–276 °C [46].

^1^HNMR (400 MHz, CDCl_3_, δ): 9.38–9.35 (m, 2H), 8,59–8.57 (d, 2H), 8.53 (d, 1H), 8.21–8.19 (d, 1H), 7.94–7.91 (m, 1H), 7.85–7.74 (m, 4H)

^13^C NMR (100 MHz, CDCl_3_, δ): 133.7, 132.3, 132.26, 131.2, 131.0, 130.9, 130.2, 128.2, 128.1, 126.6, 126.6, 123.0, 123.0

*12-Bromo-dibenzo[f, h]pyrido[2, 3-b]quinoxaline-FN10*

5-bromo-1,2-diaminobenzene (0.898 g, 4.8 mmol) was refluxed with 9,10-phenanthrenequinone (1 g, 4.8 mmol) in glacial acetic acid (80 mL) for 2 h. The compound was obtained as yellow crystals: C_19_H_10_BrN_3_, 360.21 g/mol, 1.42 g, yield 77%, m.p. 235–238 °C, lit. 235–238 °C [49,57].

^1^HNMR (400 MHz, CDCl_3_, δ): 9.43–9.41 (d, 1H), 9.25 (s, 1H), 9.20–9.18 (d, 1H), 8.77 (s, 1H), 8.52–8.50 (d, 2H), 7.85–7.68 (m, 4H)

^13^C NMR (100 MHz, CDCl_3_, δ): 155.3, 148.0, 145.1, 144.1, 139.5, 137.4, 132.5, 132.5, 131.4, 131.2, 129.4, 129.1, 128.3, 128.1, 127.4, 126.6, 123.0, 122.9, 120.5

*Dibenzo[f, h]pyrazino[2,3-b]quinoxaline-FN11*

5-bromo-2,3-diaminopyrazine (0.9 g, 4.8 mmol) was refluxed with 9,10-phenanthrenequinone (1 g, 4.8 mmol) in glacial acetic acid (80 mL) for 2 h. The compound was obtained as yellow crystals: C_18_H_9_BrN_4_, 361.21 g/mol, 1.19 g, yield 69%, m.p. 279–281 °C.

^1^HNMR (400 MHz, CDCl_3_, δ): 9.56 (d, 1H), 8.22–8.19 (d, 2H), 8.04–8.02 (d, 2H), 7.76–7.72 (m, 2H), 7.51–7.47 (m, 2H)

^13^C NMR (100 MHz, CDCl_3_, δ): 180.3, 135.9, 135.8, 131.0, 130.5, 129.9, 129.5, 129.5, 123.9, 123.6

*Dipirydo[3,2-a:2′,3′-c]phenazine-FN12*

2,3-diaminobenzene (1.0 g, 9.2 mmol) was refluxed with 1,10-phenanthroline-5,6-dione (2 g, 9.2 mmol) in glacial acetic acid (150 mL) for 2 h. The compound was obtained as yellow crystals: C_18_H_10_N_4_, 282.30 g/mol, 1.74 g, yield 65%, m.p. 248–250 °C, lit. 254–255 °C [61], 248–250 °C [57].

^1^HNMR (400 MHz, CDCl_3_, δ): 9.71 (d, 2H), 9.33–9.32 (d, 2H), 8.40–8.38 (m, 2H), 7.97–7.95 (m, 2H), 7.86–7.83 (m, 2H)

^13^C NMR (100 MHz, CDCl_3_, δ): 175.7, 152.4, 148.2, 142.5, 141.1, 133.9, 130.7, 129.5, 127.6, 124.2

*Phenanthro[9, 10-g]pteridine-FN13*

4,5-diaminopyrimidine (1.0 g, 9.1 mmol) was refluxed with 9,10-phenanthrenequinone (1.9 g, 9.1 mmol) in glacial acetic acid (150 mL) for 2 h. The compound was obtained as yellow crystals: C_18_H_10_N_4_, 282.30 g/mol, 0.96 g, yield 71%, m.p. 320–321 °C.

^1^HNMR (400 MHz, DMSO-*d*_6_, δ): 9.55–9.52 (d, 2H), 9.22–9.21 (d, 2H), 8.41–8.37 (d, 2H), 8.08–8.04 (d, 2H), 7.96–7.93 (d, 2H)

^13^C NMR (100 MHz, DMSO-*d*_6_, δ): 172.4, 152.8, 148.3, 142.2, 141.3, 133.5, 131.7, 129.6, 127.4, 125.0

### 2.3. Methods

The ^1^H NMR (400 MHz) spectra were recorded on Bruker Ascend^TM^ 400 NMR spectrometers, Billerica, MA, United States. Deuterated chloroform (CDCl_3_) and dimethylsulfoxide (DMSO-*d6*) was used as the solvent. Chemical shifts are reported in ppm relative to internal tetramethylsilane (TMS) reference, and coupling constants are reported in Hertz. Splitting patterns are described as singlet (s), doublet (d), triplet (t), quartet (q), or multiplet (m).

The melting points (uncorrected) were determined with the use of a Boëthius apparatus, Vernon Hills, IL, United States.

For thin layer chromatography, Merck Co, Kenilworth, NJ, United States., silica gel 60 F254 sheets (0.2 mm thickness) and chloroform as an eluent were used.

The electronic absorption (c ≈ 10^−4^–10^−3^ M) and fluorescence spectra in ethyl acetate (EtOAc) were recorded at room temperature using Shimadzu UV-1280, Kioto, Japan and Hitachi F-7100 spectrophotometers, Tokio, Japan, respectively. The fluorescence quantum yield (FQY) was determined by a comparative method [62] using Coumarin 1 in ethanol (*φ_ref_* = 0.64 [63]) as the reference. The absorbances of both the dye and reference solution at an excitation wavelength (366 nm or 404 nm) was ca. 0.1.

The nanosecond laser flash photolysis experiments were performed using LKS.60 Laser Flash Photolysis apparatus (Applied Photophisics, Leatherhead, United Kingdom). Laser irradiation at 355 nm from the third harmonic of the Q-switched Nd:YAG laser from a Lambda Phisik/model LPY 150 operating at 65 mJ/pulse (pulse width about 4–5 ns) was used for the excitation. Transient absorbances at preselected wavelengths were monitored by a detection system consisting of a monochromator, a photomultiplier tube (Hamamatsu R955), and a pulsed xenon lamp (150 W) as a monitoring source. The signal from the photomultiplier was processed by a Hewlett-Packard/Agilent an Agilent Infiniium 54810A digital storage oscilloscope and an Acorn-compatible computer.

**Scheme 1:**
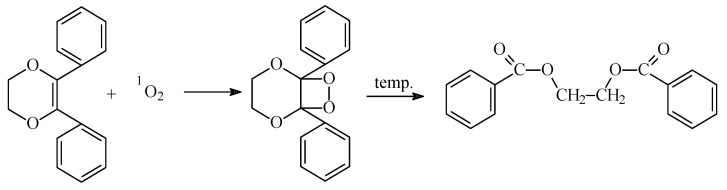
Oxidation of 2,3-diphenyl-*p*-dioxene with singlet oxygen.

The quantum yield of the triplet state formation of the synthesized dyes was determined by two methods. The first one was described by Lament et al. [64] and was based on the analysis of transient absorption spectra, whereas the second one was described by Silverman and Foote [32]. The latter method was based on the analysis of ^1^H NMR spectra recorded during the singlet oxygen oxidation of 2,3-diphenyl-*p*-dioxene (Scheme 1). The photooxidation reaction was carried out in a solution containing 0.6 mL of deuterated chloroform in which both 10 mg of 2,3-diphenyl-*p*-dioxene and 5 mg of the dye were dissolved. The solution was irradiated with LED F (dental-lamp-emitted) radiation in the range: 390–480 nm. The radiation intensity was 18 mW/cm^2^. During irradiation, the solution was stirred with a stream of oxygen. The formation of the photooxidation product was monitored by recording the ^1^H NMR spectra.

The quantum yield of singlet oxygen production was calculated based on the rate of the ethylene glycol dibenzoate formation by the tested sensitizers compared to the rate of this product’s formation in the presence of camphorquinone, which was used as a standard. The quantum yield of singlet oxygen generation by camphorquinone was 1.0 [65,66]. With a large excess of oxygen, the quantum yield of singlet oxygen formation was equal to the quantum yield of the triplet state formation [24].

(5) ΦO21=ΦT

The effectiveness of the synthesized dyes as radical polymerization photosensitizers was tested by the microcalorimetric method [67,68]. The measurements were performed in a homemade microcalorimeter using a semiconducting diode immersed in a 2–3 mm thick layer (0.25 mL) of a cured sample as a temperature sensor. The initial rate of polymerization was determined by the measurements of the rate of the heat evolution during polymerization for the initial time. A diode-pumped solid-state laser (408 nm) with an intensity of 18 mW/cm^2^ (Mean Well model NES-15-12) was used as the light source. The light intensity was measured by a Coherent model Fieldmaster power meter. The distance of the sample from the light source was the same for all measurements. Each measurement was repeated at least three times.

The photopolymerization compositions were composed of 0.9 g of trimethylolpropane triacrylate (TMPTA) and 0.1 mL of 1-methyl-2-pyrrolidinone (MP) containing appropriate amounts of the photosensitizer (synthesized phenazine based dyes) and the co-initiator [(3,4-dimethoxyphenyl)sulfanyl]acetic acid (MKTFO). The concentration of the dyes was 2 × 10^−3^–5.63 × 10^−4^ M (depending on the molar absorption coefficient at an excitation wavelength), whereas the concentration of the co-initiator was 0.1 M. As a reference sample, a polymerizing mixture without a co-initiator was used.

In order to compare the quantum efficiency of triplet state formation and the photoinitiating efficiency of the TMPTA, radical polymerization by the synthesized compounds with a commercial compound, the camphorquinone (λ_max_ = 472 nm, ε = 40 M^−1^cm^‒1^ in ethyl acetate), was used. Its concentration in the polymerization experiments was 0.675 M.

## 3. Results

**Figure 1:**
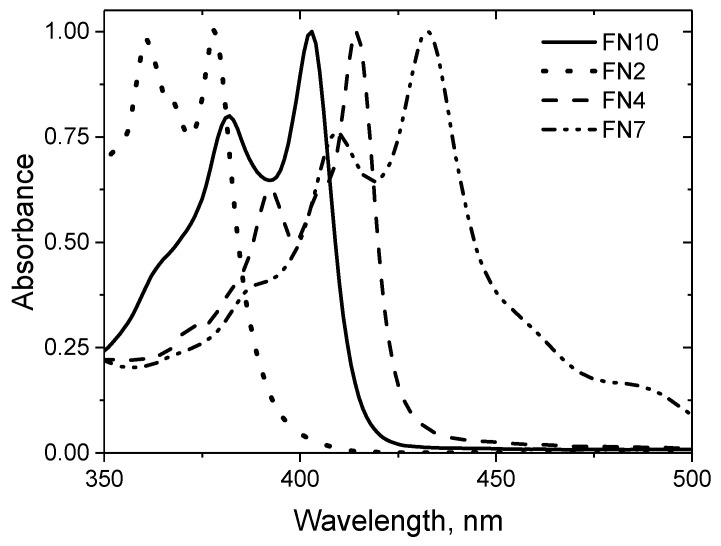
Fragments of the electronic absorption spectra of the selected dyes in ethyl acetate.

The analysis of the electronic absorption spectra of the synthesized compounds indicated that the spectra were typical for heterocyclic aromatic compounds with condensed rings and that they were characterized by several absorption maxima in the long-wavelength region (Figure 1). They displayed main absorption bands in the range from 350 nm to 530 nm. Thus, the compounds afforded deeply colored solutions with molar extinction coefficients ranging from 0.96 × 10^4^ M^−1^cm^−1^ to 2.56 × 10^4^ M^−1^cm^−1^ for the most intense band, which is typical for π→π* transitions. The basic spectral data are collected in Table 1.

The position of the absorption bands was clearly influenced by the dye structure, i.e., the number of conjugated double bonds, the arrangement of aromatic rings, and the number and location of nitrogen atoms. The increase in the number of conjugated double bonds in the molecule (FN1, FN2, and FN3; FN6 and FN7) and the angular arrangement of the rings (FN4 versus FN3) caused a bathochromic effect. The red shift of the absorption maximum was also observed with an increase in the number of nitrogen atoms in the molecule (FN9, FN10, FN11) as well as the presence of a heavy atom (FN2 and FN9) (Figure S1 in ESI).

In general, the compounds had high molar absorption coefficients and appropriate spectral characteristics enabling selective excitation of the sensitizer. It follows that the dyes met the requirements for singlet oxygen sensitizers.

**Figure 2:**
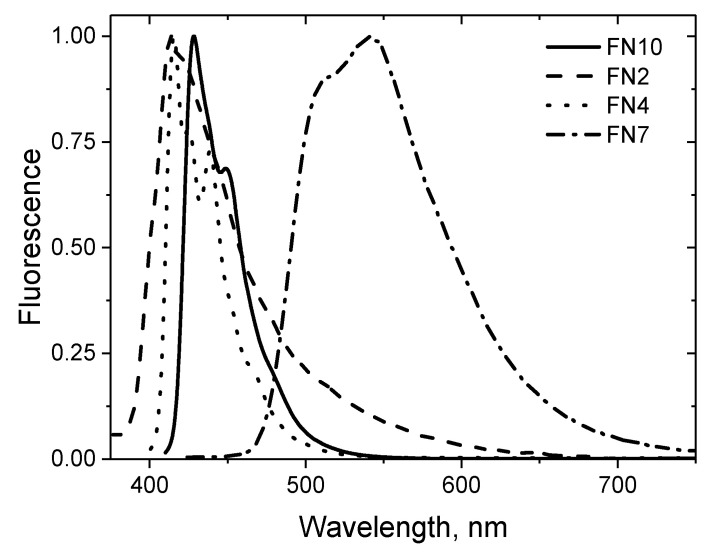
Fluorescence spectra of the selected dyes in ethyl acetate.

Figure 2 shows the fluorescence spectra for selected dyes.

As for absorption spectra, the position of the fluorescence maximum depended on the dye structure. The number of both conjugated double bonds and nitrogen atoms in the molecule, the angular arrangement of the rings, and the presence of a heavy atom affected the fluorescence band position and its intensity (Figure S2 in ESI). The data in Table 1 show that the fluorescence quantum yield varied from 0.025 (FN6 and FN7) for compounds with a high singlet-oxygen-generating ability to 0.11 (FN1) for the compound showing a very low singlet oxygen quantum yield (Table 2).

**Figure 3:**
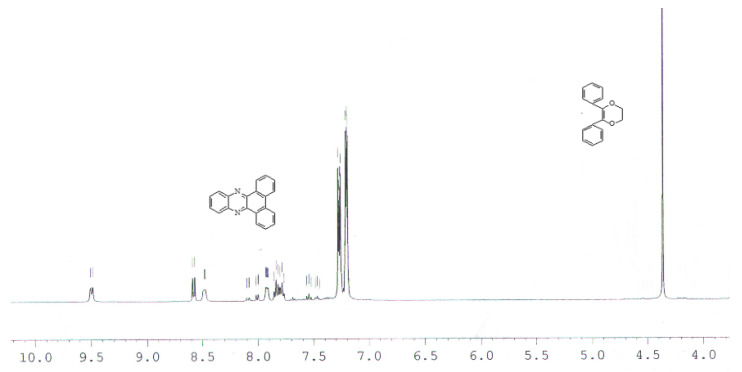
^1^H NMR spectrum of a mixture of dibenzo[a.c]phenazine sensitizer and 2,3-diphenyl-*p*-dioxene in CDCl_3_ before irradiation.

**Figure 4:**
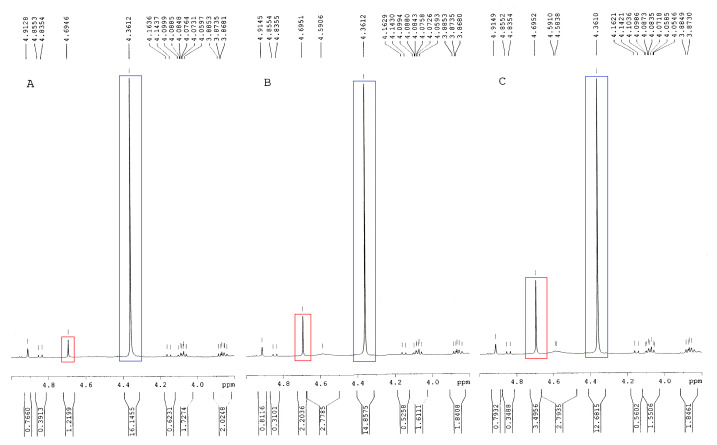
^1^H NMR spectra of a mixture of dibenzo[a.c]phenazine (sensitizer) and 2,3-diphenyl-*p*-dioxene in CDCl_3_; irradiation time: A-10 min, B-20 min, and C-30 min.

The quantum yield of the triplet state formation was determined by the comparative method using camphorquinone as a standard. The method was based on the oxidation of 2,3-diphenyl-*p*-dioxene by singlet oxygen produced in a photochemical process involving the excitation of triplet oxygen with UV–vis light in the presence of a sensitizing dye. The formation of the photooxidation product was monitored by the analysis of the ^1^H NMR spectra recorded for the initial solution and after different times of irradiation (Figure 3 and Figure 4).

The ^1^H NMR spectra of the tested compounds in the presence of 2,3-diphenyl-*p*-dioxene in CDCl_3_, recorded after different irradiation times, showed the formation of photooxidation products according to Equation (5) (Figure 3 and Figure S3 in ESI). Their analysis allowed us to distinguish the substrate from the oxidation product, for which the relevant values of characteristic chemical proton shifts are presented in Table 3.

The analysis of ^1^H NMR spectra of the tested compounds in a mixture with 2,3-diphenyl-*p*-dioxene in CDCl_3_ indicated a well-separated singlet for two methylene groups from the 2,3-diphenyl-*p*-dioxene. After gradual irradiation, the proton spectrum of the same solution showed considerable changes in chemical shifts. It became evident that the signal intensity at 4.3612 ppm decreased gradually accompanied by the appearance of a new signal at 4.5916 ppm. The intensity of the new singlet increased proportionally with the irradiation time. The location of the signal was in perfect agreement with the two methylene groups in ethylene glycol dibenzoate, which was formed during photooxidation of the 2,3-diphenyl-*p*-dioxene sensitized by the tested compounds. The quantum yield of singlet oxygen production in the presence of the tested sensitizers is collected in Table 3. These values can be considered equal to the quantum yield of the triplet state formation when determined under the conditions of excess oxygen.

**Figure 5:**
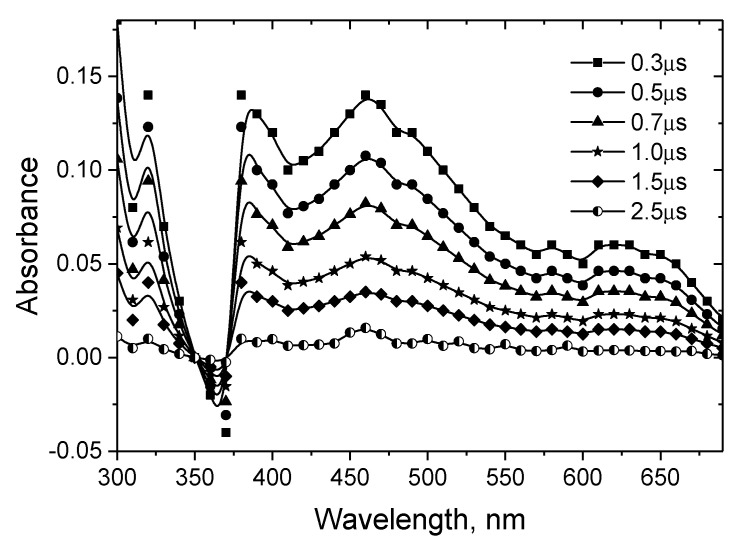
Laser flash photolysis spectra of the dye FN2 in acetonitrile recorded after irradiation with laser pulses of 355 nm. Delay times shown in legends.

The quantum yield of the triplet state formation for selected sensitizers was also determined from the analysis of transient absorption spectra obtained using the nanosecond laser flash photolysis technique. An exemplary spectrum of FN2 in deoxygenated acetonitrile solution is shown in Figure 5. A readily detectable transient absorption spectra peaking at 390 nm, 470 nm, and 620 nm decay in a few milliseconds according to first-order kinetics. They were not observed in an oxygen-saturated solution.

**Figure 6:**
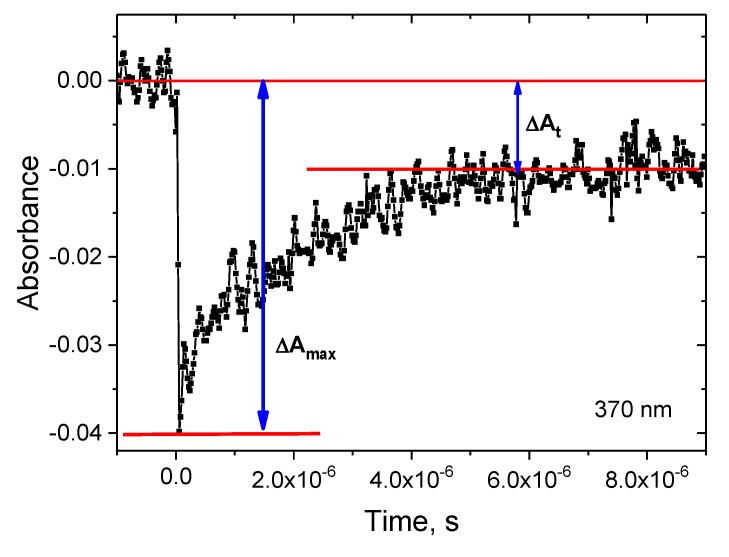
Analysis of ΔA(t) curve recorded for FN2 in acetonitrile at 370 nm.

The quantum yields of the triplet state formation of the tested sensitizers were determined from the triplet formation curves using methodology given by Lament et al. [64] and by us [36]. The method is based on quantitative analysis of the ground state recovery and its deconvolution, which concerns an instrumental response function. A typical example of such approach is shown in Figure 5, and obtained data are summarized in Table 2. According to Equation (6), the yield of triplet formation (Φ_T_) is proportional to the ratio of the initial increment in absorbance immediately after the flash (ΔA_t_) and the value at the maximum ground state depletion (the former is compared to the long delay, “plateau” region) (cf. Figure 6):

(6) $$\Phi_{T}=\frac{\Delta A_{t}}{{\Delta A}_{max}}$$

**Figure 7:**
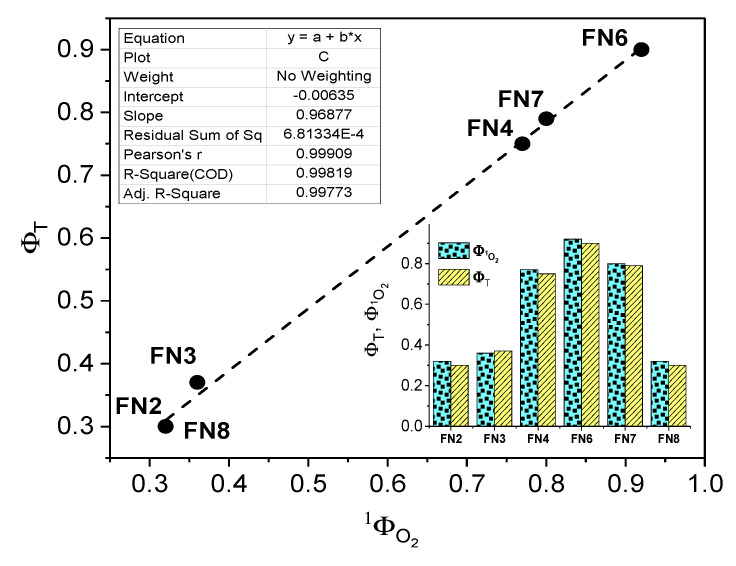
Comparison of the quantum yield of singlet oxygen generation to the quantum yield of triplet state formation for selected sensitizers.

The comparison of the data collected in Table 2 and illustrated in Figure 7 shows unequivocally the equivalence of the methods for assessing the quantum yield of the triplet state formation and the validity of Equation (5).

From the viewpoint of the photochemical properties of the tested compounds, the data presented in Table 2 indicate that the structure of the sensitizer tested had a significant influence on the ability to generate singlet oxygen and, thus, to form a triplet state.

Introduction of a bromide atom to the phenazine scaffold (dye FN2 vs. FN9) increased the ability to generate singlet oxygen in the photochemical process. Furthermore, the quantum yields of triplet state formation decreased from about 0.67 to 0.51 and to 0.31 for dyes containing two (FN9), three (FN10), and four (FN11) nitrogen atoms, respectively. This proves that the modification of the phenazine structure by introducing more nitrogen atoms into the molecule leads to a reduction in the ability of the compounds to sensitize singlet oxygen. The ability to generate singlet oxygen by the phenazine derivatives was also influenced by the number of conjugated double bonds and the arrangement of aromatic rings (linear and/or angular system). The highest quantum yield of singlet oxygen production was characterized by phenazine derivatives with angular arrangements of the aromatic rings (FN4 compared to FN3 and FN6 compared to FN 7).

**Figure 8:**
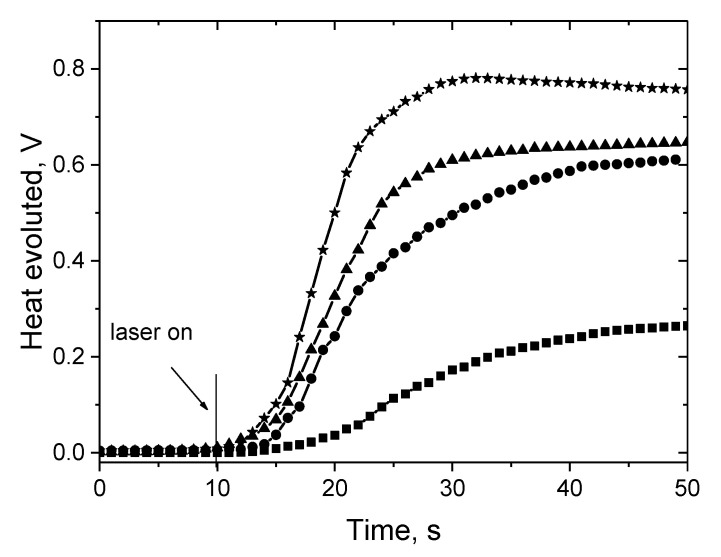
Family of kinetic curves recorded during the measurements of the heat flow emitted during the photoinitiated polymerization of the TMPTA-MP mixture initiated by tested dyes—[(3,4-dimethoxyphenyl)sulfanyl]acetic acid couples. The co-initiator concentration was 0.1 M, and the light intensity of the diode laser (408 nm) was 18 mW/cm^2^. The applied dyes possessed various chromophores: ⁎—FN4, ▲—FN2, ●—FN3, ■—FN1.

**Figure 9:**
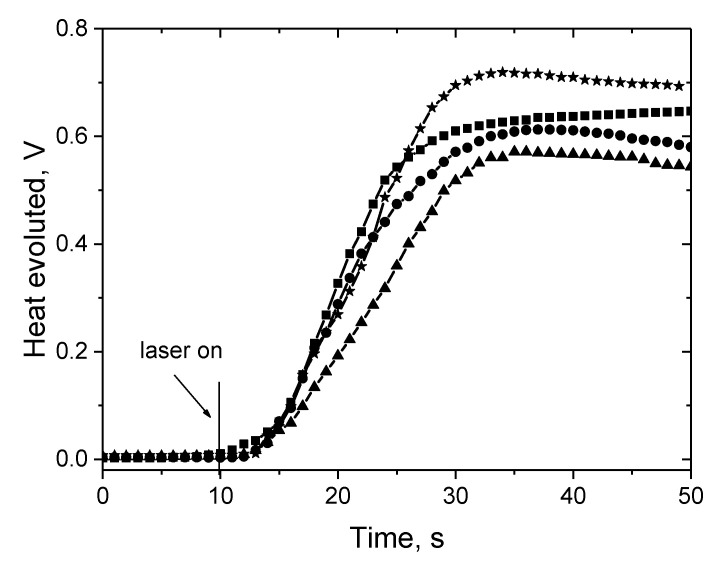
Family of kinetic curves recorded during the measurements of the heat flow emitted during the photoinitiated polymerization of the TMPTA-MP mixture initiated by tested dyes—[(3,4-dimethoxyphenyl)sulfanyl]acetic acid couples. The co-initiator concentration was 0.1 M, and the light intensity of the diode laser (408 nm) was 18 mW/cm^2^. The applied dyes possessed various chromophores: ■—FN2, ⁎—FN12, ●—FN8, ▲—FN13.

Since the quantum yield of singlet oxygen generation at a high excess of oxygen translates into the quantum yield of the triplet state formation (which is a desired feature of photoinitiators), the obtained sensitizers were used to initiate the TMPTA polymerization. According to Equation (7) [67,68], the rate of photoinitiated radical polymerization is directly proportional to the square root of the quantum yield of triplet state formation:

(7) $$R_{p}=-\frac{d\left[M\right]}{\mathrm{dt}}=k_{p}\left[M\right]\sqrt{\frac{I_{a}\Phi_{T}}{k_{t}}}$$

where:

*M*—the molar concentration of the monomer;
*k_p_*—the polymerization rate constant;
*I_a_*—the absorbed radiation intensity;
*φ_T_*—the quantum yield of triplet state formation;
*k_t_*—the macroradical termination rate constant;

The efficiency of the polymerization initiated by phenazine dye-[(3,4-dimethoxyphenyl)sulfanyl]acetic acid (MKTFO) systems was assessed from the heat flow during the visible light irradiation. Typical kinetic curves obtained for the photoinitiated polymerization of the TMPTA-MP (9:1) mixture recorded for selected dyes are shown in Figure 8 and Figure 9 for illustration. The rates of photoinitiated polymerization determined for all the tested photoredox pairs are collected in Table 2.

The analysis of the presented curves and the *R_p_* values collected in Table 2 indicated that the highest efficiency of photoinitiating polymerization was characterized by those phenazine derivatives for which the quantum yields of singlet oxygen generation (formation of a triplet state) were the highest.

**Figure 10:**
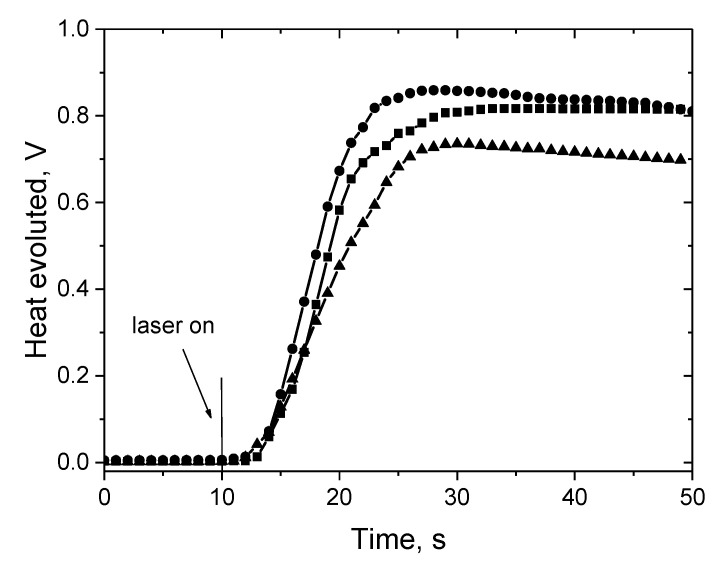
Family of kinetic curves recorded during polymerization of TMPTA-MP photoinitiated with ●—CQ, ■—FN7, ▲—FN9 in the presence of the MKTFO co-initiator (0.1 M); the light intensity of diode laser (408 nm) was 18 mW/cm^2^.

The effectiveness of the synthesized dyes in the radical polymerization photoinitiation, characterized by the initial rate *R_p_*, was compared to the camphorquinone—a commercial photoinitiator used in dentistry (Figure 10).

The results clearly indicated that the initial rate of radical polymerization photoinitiated by phenazine derivatives was comparable to the polymerization rate in which commercial CQ was used as a photoinitiator (Figure 10). However, the concentration of the synthesized dyes in the polymerizing composition was about 2000 times lower than the CQ. The CQ molar absorption coefficient at the excitation wavelength (λ_max_ = 408 nm) was only ε = 8 M^−1^cm^−1^, whereas for the synthesized dyes, e.g., FN9 (λ_max_ = 408 nm), it was ε = 15,400 M^−1^cm^−1^. This made it possible to obtain thick polymer layers (4 mm) that did not contain significant amounts (about 0.675 M) of unreacted CQ and co-products formed during the photochemical reaction (the radical resulting from the dye usually does not initiate a chain reaction but participates in side reactions).

**Figure 11:**
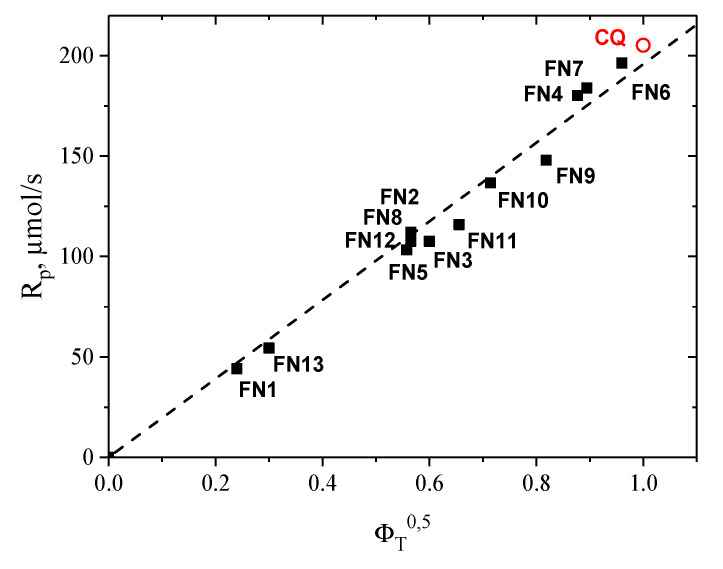
Correlation between the rate of photoinitiated polymerization of TMPTA and the square root of the quantum yield of the triplet state formation for FN1–FN13 and ○–CQ in the presence of MKTFO co-initiator (0.1 M); the light intensity of diode laser (408 nm) was 18 mW/cm^2^.

According to Equation (7), the radicals’ formation takes place in the triplet excited state of the dye. If this assumption is correct for the tested photoinitiator systems, the initial rate (*R_p_*) of TMPTA photoinitiated polymerization should depend linearly on the square root of the quantum yield of the triplet state. On the basis of the correlation shown in Figure 11, it can be concluded that the electron transfer process from the electron donor (MKTFO) to the photosensitizers (FN1–FN13) occured in the triplet excited state.

## 4. Conclusions

The obtained results show that phenazine derivatives are interesting and efficient singlet oxygen sensitizers. In some cases, the quantum yield of generating singlet oxygen was close to 1.0. Increasing the number of nitrogen atoms in the modified phenazine structures clearly decreased the quantum yield of the triplet state formation. This proves that the modified phenazine structures containing large number of nitrogen atoms are weak singlet oxygen sensitizers. In addition, the ability to generate singlet oxygen by the modified phenazine structures was also influenced by the number of conjugated double bonds and the arrangement of aromatic rings. Angular arrangement of aromatic rings in the structure of phenazine derivatives guarantees obtaining good singlet oxygen sensitizers.

In conditions of high oxygen excess, the quantum yield of singlet oxygen generation translates into the quantum yield of the triplet state formation, which allows the obtained photosensitizers to be used in the TMPTA radical polymerization process. The initiation efficiency of TMPTA polymerization increases with the increase in the quantum yield of the singlet oxygen production and, thus, the quantum yield of the triplet state formation.

The greatest application potential reveals compounds having a heavy atom or an angular arrangement of the rings. The analysis of the data described in this study and in our previous articles [69,70,71] leads to the conclusion that the appropriate modification of the dye structure allowed the obtainment of compounds that are not only effective photosensitizers in systems initiating radical polymerization but also sensitizers of singlet oxygen.

## Figures and Tables

**Table 1:** Structures, basic spectroscopic properties, and quantum yields of fluorescence for the dyes tested.

Dye	Structure	$\lambda_{max}^{Ab},\;nm$	$\epsilon_{max}^{Ab},\;M^{-1}cm^{-1}$	$\lambda_{max}^{Fl},\;nm$	$\Phi_{Fl}$
FN1	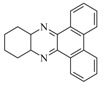	345 362	8060 10,400	383.0	0.110
FN2	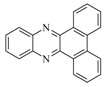	375 395	9700 11,400	413.6	0.044
FN3	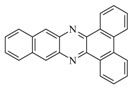	402 425	10,060 13,500	532.8	0.045
FN4	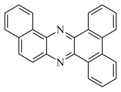	392 414	9900 15,900	415.2 438.4	0.027
FN5	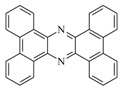	392 414	9700 16,300	414.2 438.0	0.049
FN6	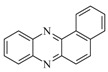	386 407	9500 10,900	437.8	0.025
FN7	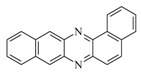	409 432	14,000 10,800	543.8	0.025
FN8	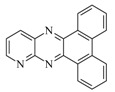	383 393	9600 9620	460	0.045
FN9	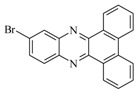	382 403	20,500 25,600	424.2	0.033
FN10	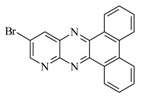	390 407	15,900 16,300	428	0.028
FN11	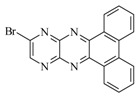	395 411	14,300 15,800	445.6	0.042
FN12	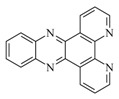	360 378	14,500 13,700	412.4	0.047
FN13	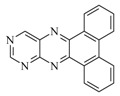	437	11,490	477	0.100
CQ	* 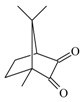 *	472	40	-	-

**Table 2:** Quantum yield of the singlet oxygen formation (ΦO21), quantum yield of the triplet state (Φ_T_), and initial rate of photoinitiated polymerization (*R_p_*) for the dyes tested.

Dye	$R_{p},\;\mu mol\;s^{-1}$	ΦO21	$\Phi_{T}$
FN1	44.1	0.06	-
FN2	112.0	0.32	0.30
FN3	107.7	0.36	0.37
FN4	180.2	0.77	0.75
FN5	101.7	0.32	-
FN6	196.3	0.92	0.90
FN7	183.9	0.80	0.79
FN8	107.6	0.32	0.30
FN9	147.9	0.67	-
FN10	136.7	0.51	-
FN11	115.9	0.31	-
FN12	103.3	0.30	-
FN13	54.4	0.09	-
CQ	234.2	1.00	-

**Table 3:** Characteristic values of chemical shifts for individual compounds and areas under the peaks after the specified irradiation time assigned from ^1^H NMR spectra.

Compound	σ, ppm	Area		
		10 min	20 min	30 min
2,3-diphenyl-*p*-dioxene	4.3612 (s, 4H)	16.1453	14.8573	12.6815
ethylene glycol dibenzoate	4.5916 (s, 4H)	1.2199	2.2026	3.4956

## Data Availability

The data is provided by Ilona Pyszka (UTP University of Science and Technology), please contact Ilona.Pyszka@utp.edu.pl. The data is not publicly available apart from the data contained in the article or Supplementary Materials.

## Supplementary Materials

The following are available online at https://www.mdpi.com/article/10.3390/ma14113085/s1, Figure S1. Normalized electronic absorption spectra of phenazine derivatives in ethyl acetate. Figure S2. Normalized fluorescence spectra of phenazine derivatives in ethyl acetate illustrating the influence of dye structure on the bands position; Ex = 366 nm or 404 nm. Figure S3. ^1^H NMR spectra of a mixture of dibenzo[a.c]phenazine FN2 (sensitizer) and 2,3-diphenyl-p-dioxene in CDCl_3_ after different irradiation time; dental lamp, 18 mW.
