# Supplementary material for: Effective Singlet Oxygen Sensitizers Based on the Phenazine Skeleton as Efficient Light Absorbers in Dye Photoinitiating Systems for Radical Polymerization of Acrylates
Source: Materials (Basel). 2021 Jun 4;14(11):3085. doi: 10.3390/ma14113085 (PMC8200244; doi:10.3390/ma14113085)
Supplement: Supplementary file 1 [file materials-14-03085-s001.zip › materials-1217604-supplementary.pdf]

Electronic Supplementary Information

Effective singlet oxygen sensitizers based on phenazine skeleton as efficient light absorbers in dye photoinitiating systems for radical polymerization of acrylates

Ilona Pyszka [1,*], Zdzisław Kucybała [1] and Beata Jędrzejewska [1,*]

[1]*UTP University of Science and Technology, Faculty of Chemical Technology and Engineering*

Seminaryjna 3, 85-326 Bydgoszcz, Poland

**Corresponding authors:*

I. Pyszka: e-mail address: Ilona.Pyszka@utp.edu.pl; Tel.: +48-52-374-9039 (IP)
B. Jędrzejewska: e-mail address: beata@utp.edu.pl; Tel.: +48-52-374-9046 (BJ)

Table of contents page

^{1}H and ^{13}C NMR spectra 2-24

Absorption spectra in ethyl acetate – Figure S1 25-26

Fluorescence spectra in ethyl acetate – Figure S2 27-28

^{1}H NMR spectra of a mixture of dibenzo[a.c]phenazine **FN2** (sensitizer) and 2,3-diphenyl-*p*-dioxene in $CDCl_3$ after different irradiation time – Figure S3 29-33

^{1}H spectrum of **FN1**

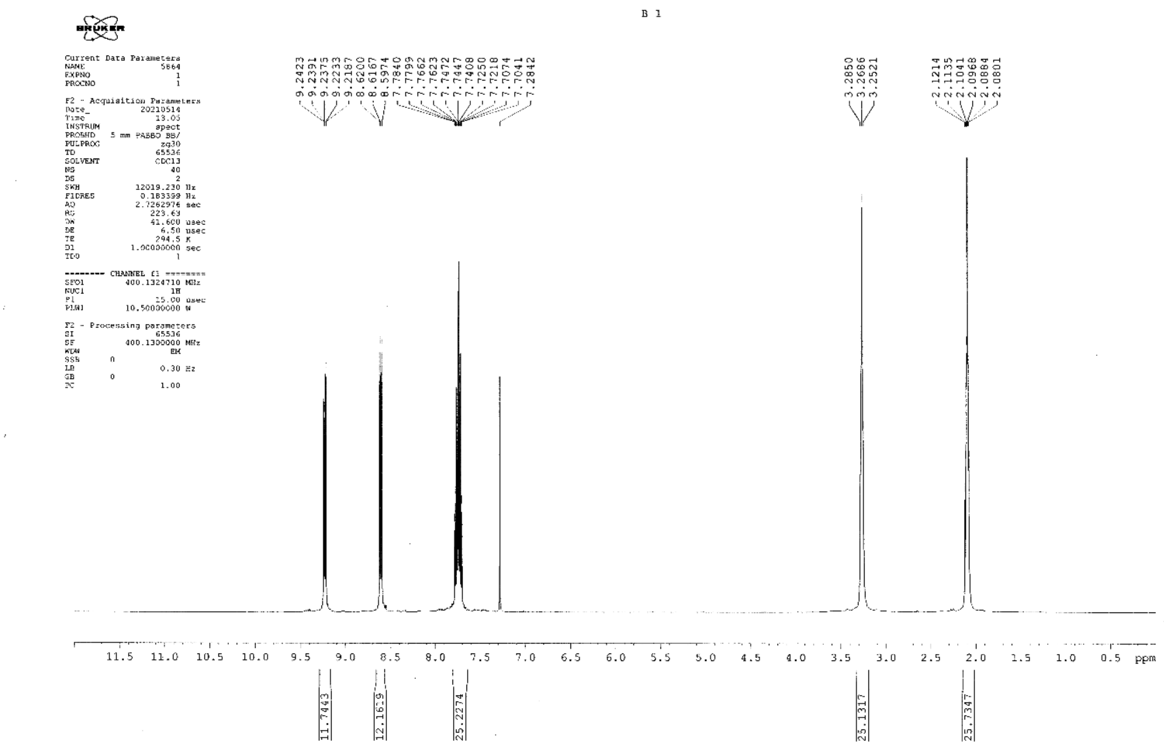

Enlarged spectrum in the range of 0-4 ppm and 7-9 ppm.

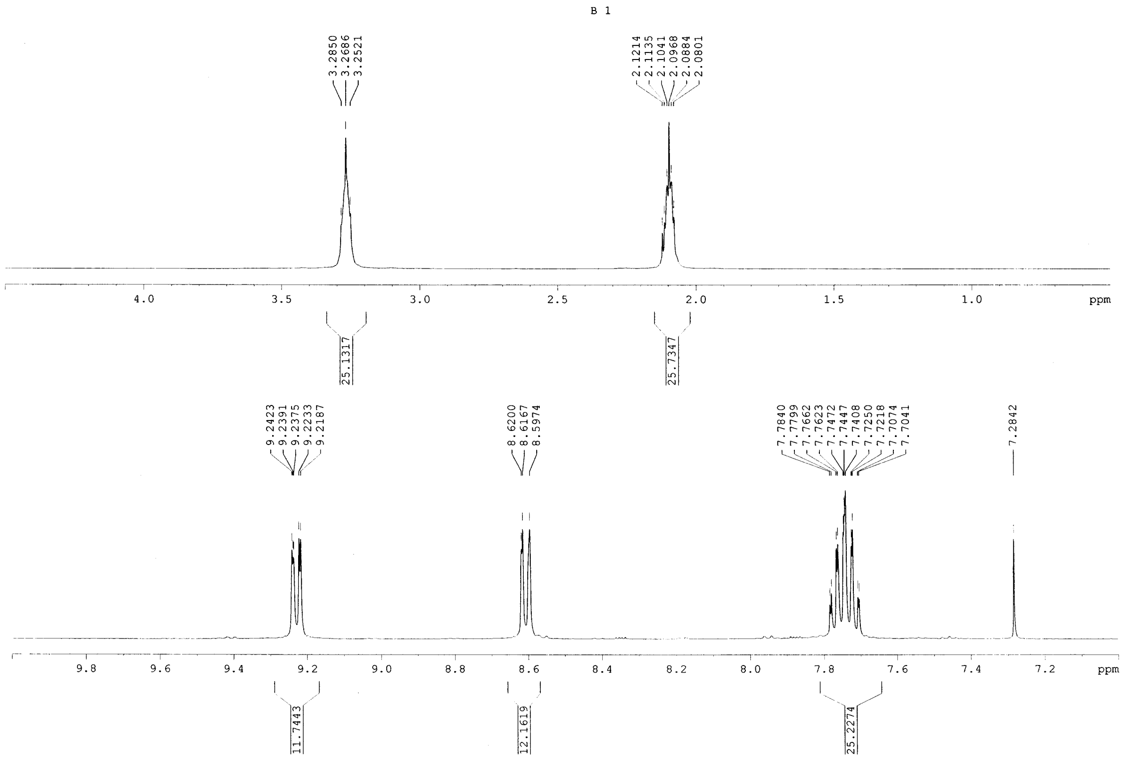

^{13}C spectrum of **FN1**

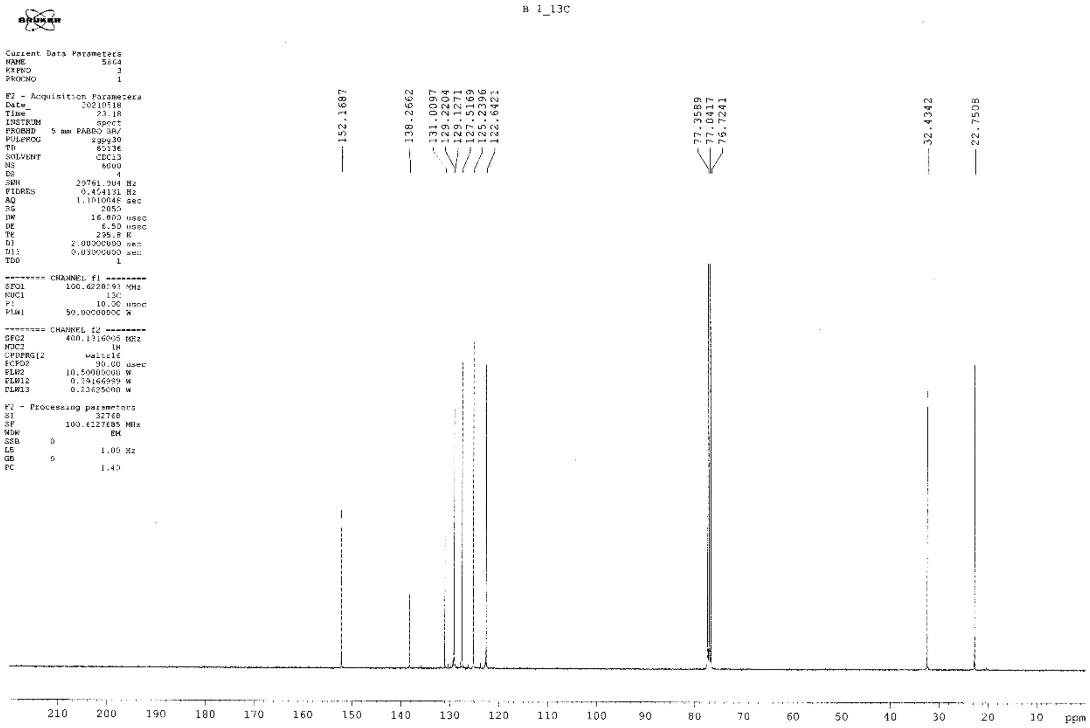

Enlarged spectrum in the range of 120-160 ppm.

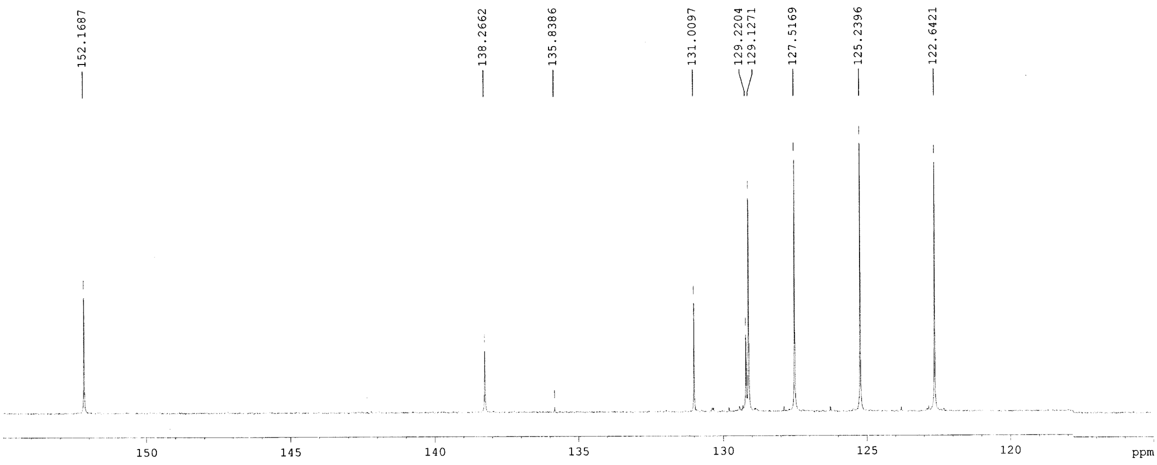

^{1}H spectrum of **FN2**

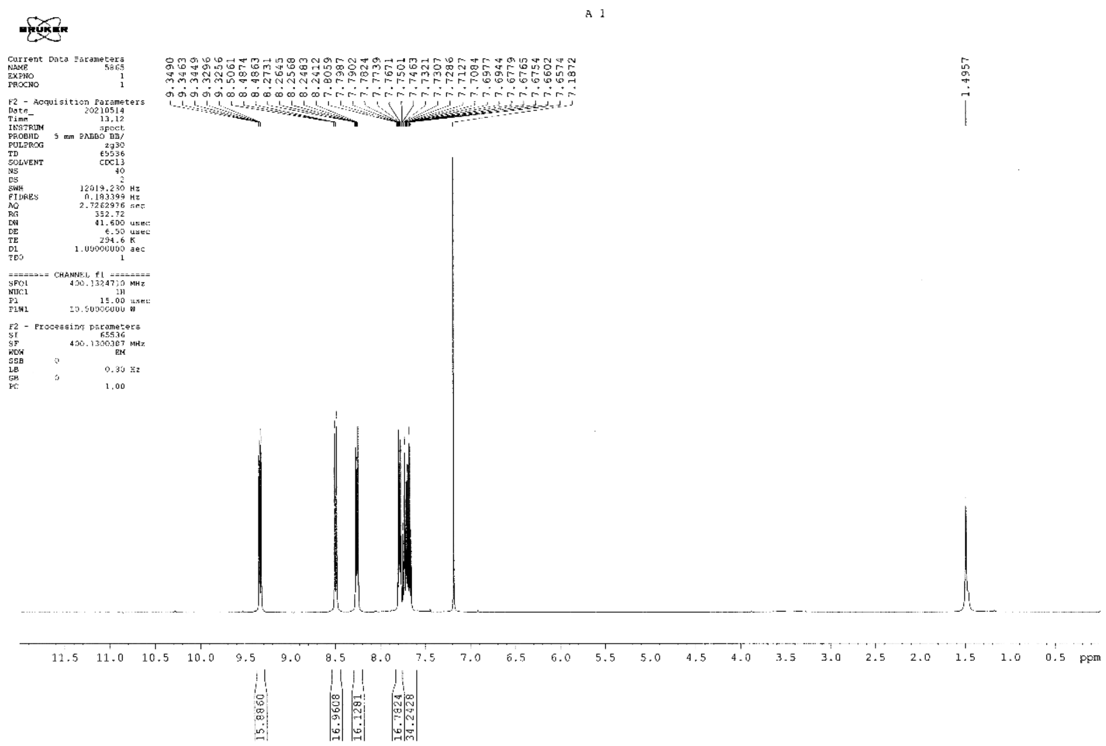

Enlarged spectrum in the range of 6-9 ppm.

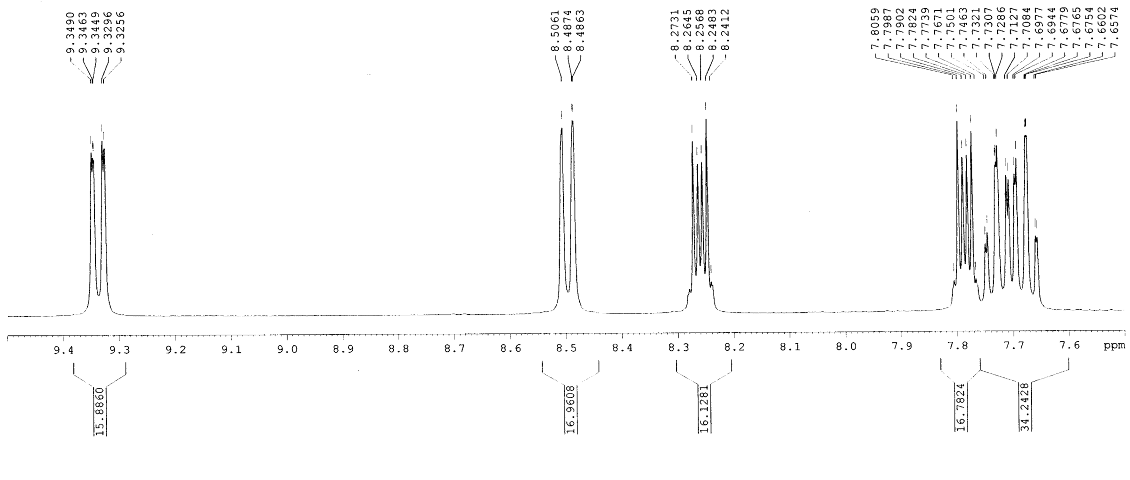

^{13}C spectrum of **FN2**

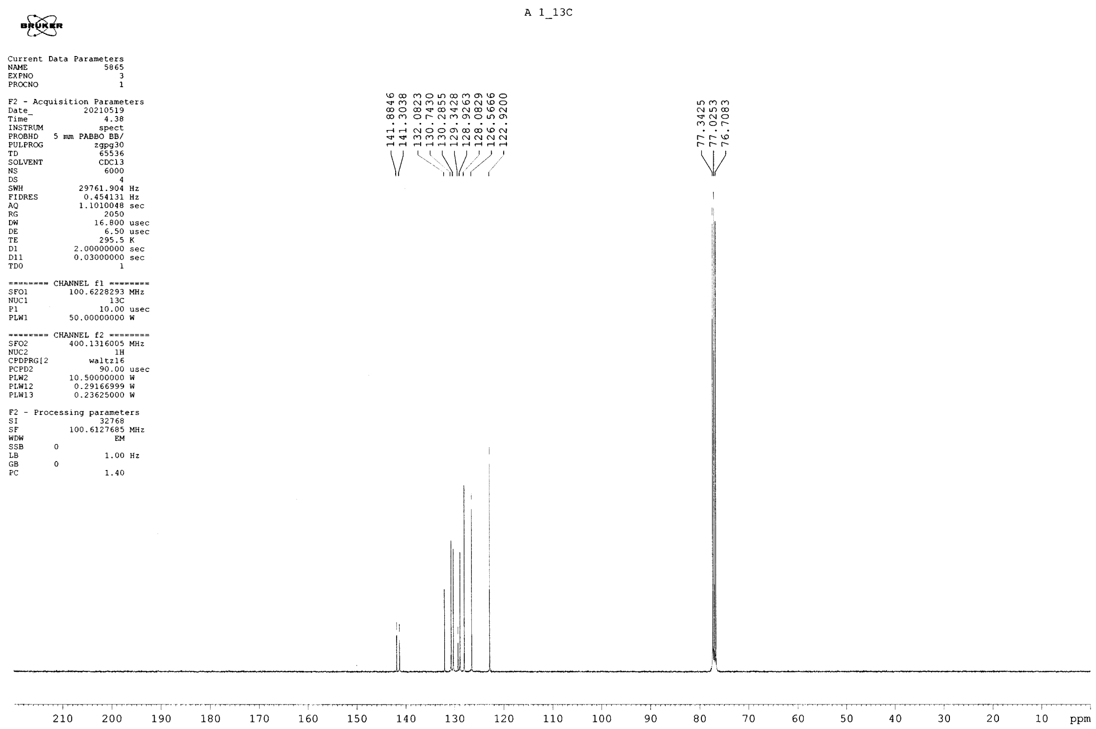

Enlarged spectrum in the range of 110-150 ppm.

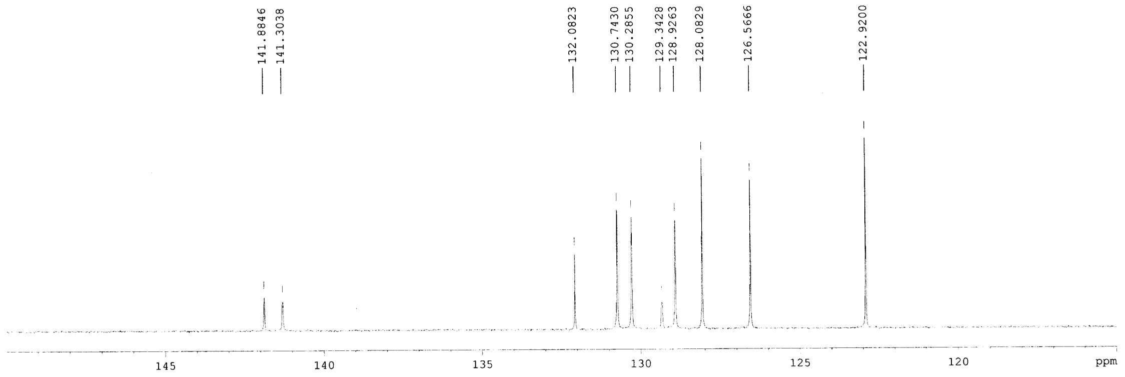

^{1}H spectrum of **FN3**

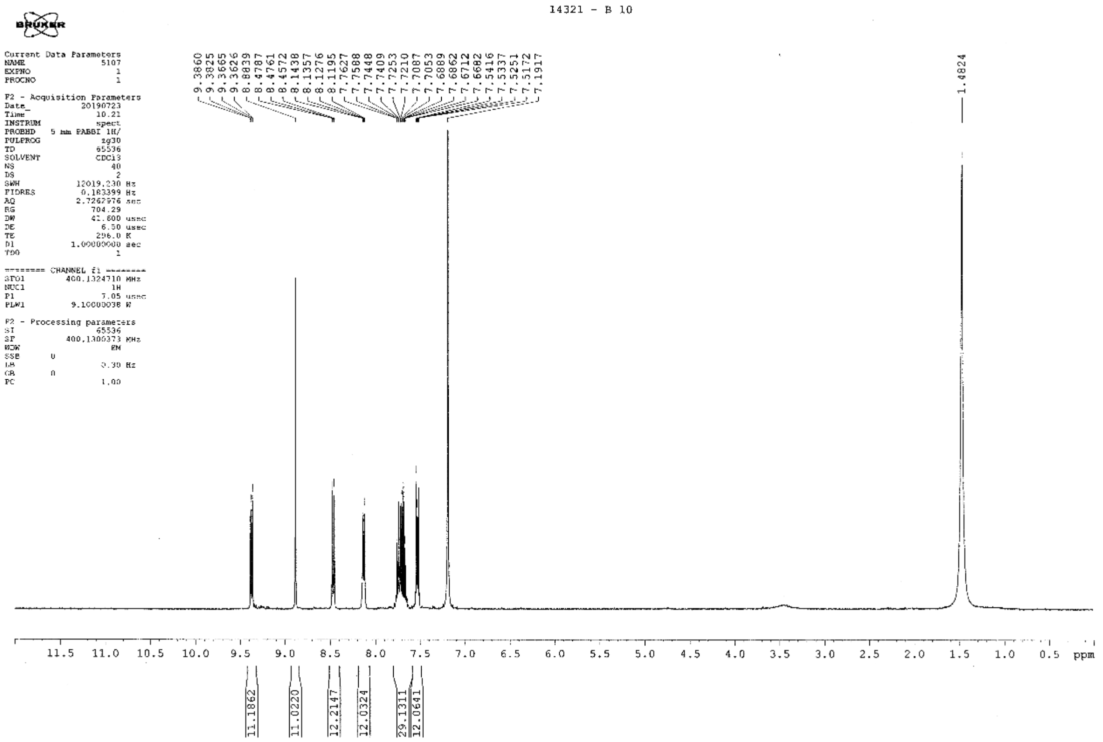

Enlarged spectrum in the range of 6-9 ppm.

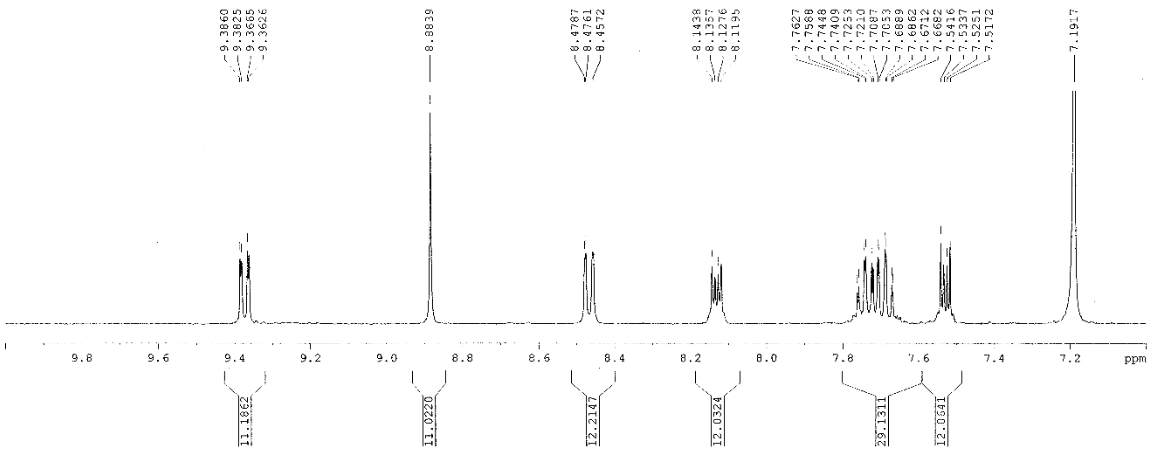

^{1}H spectrum of **FN4**

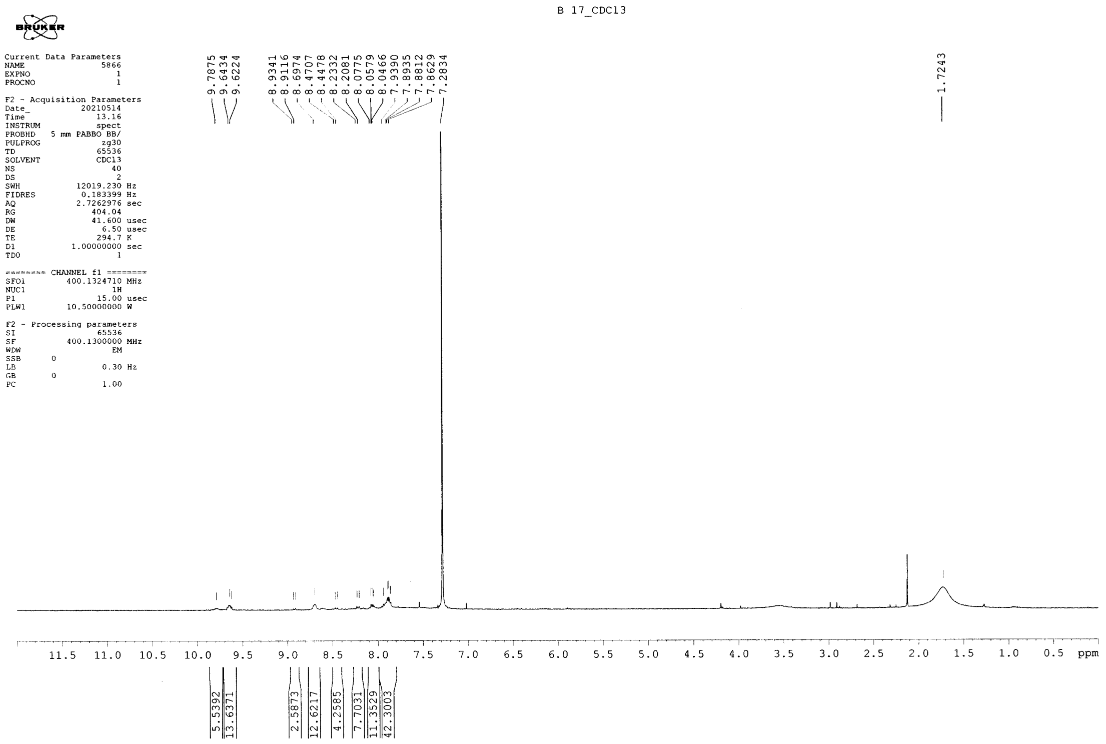

Enlarged spectrum in the range of 6-9 ppm.

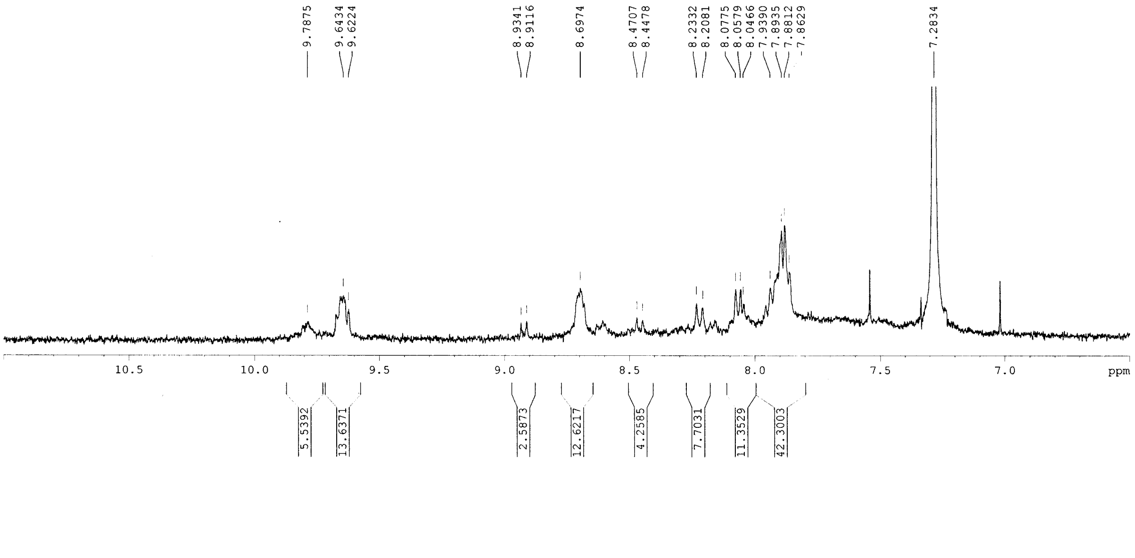

^{1}H spectrum of **FN5**

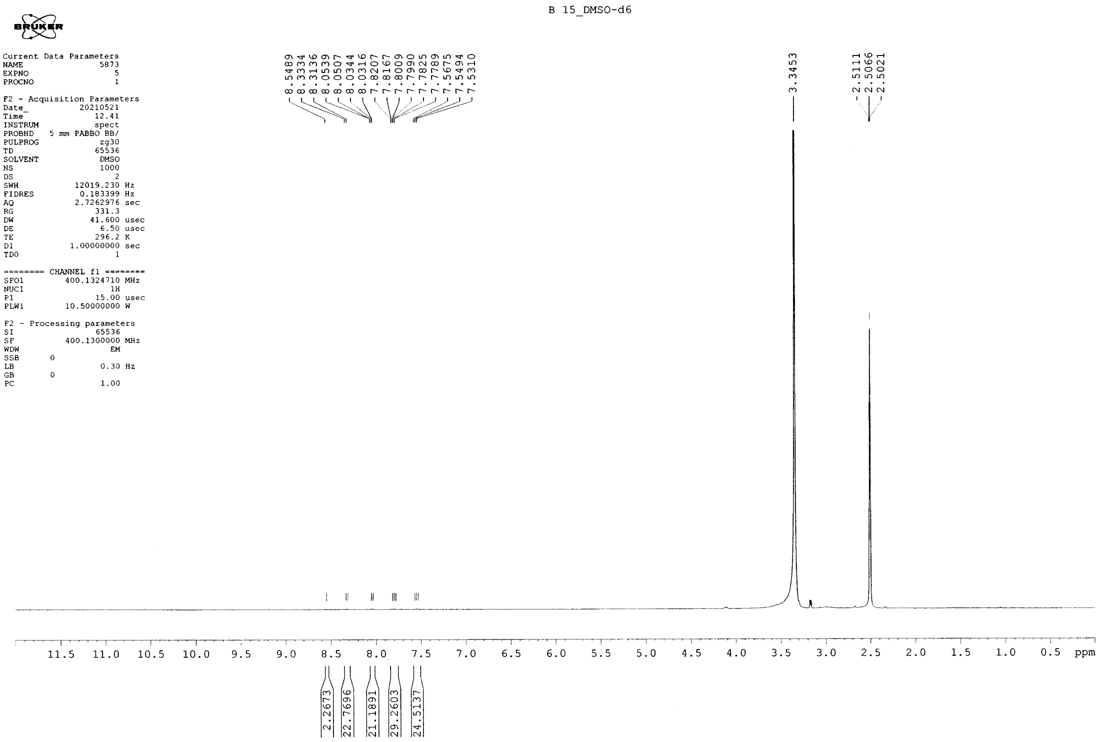

Enlarged spectrum in the range of 6-9 ppm.

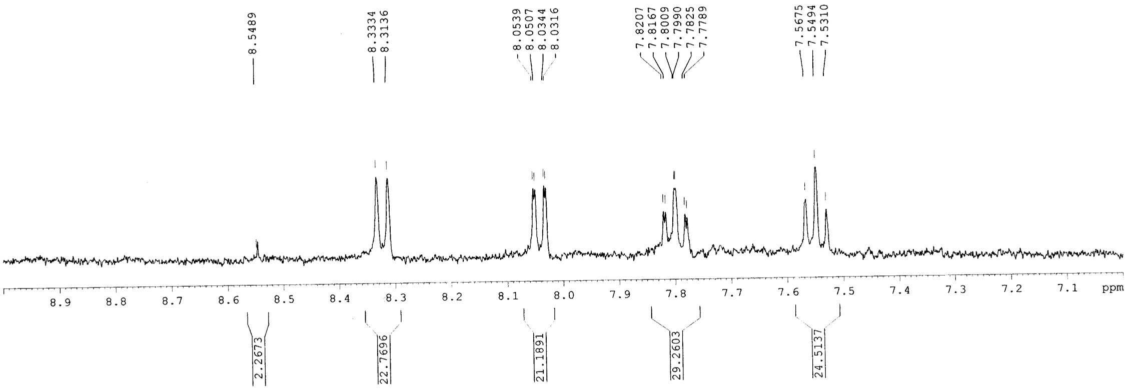

^{1}H spectrum of **FN6**

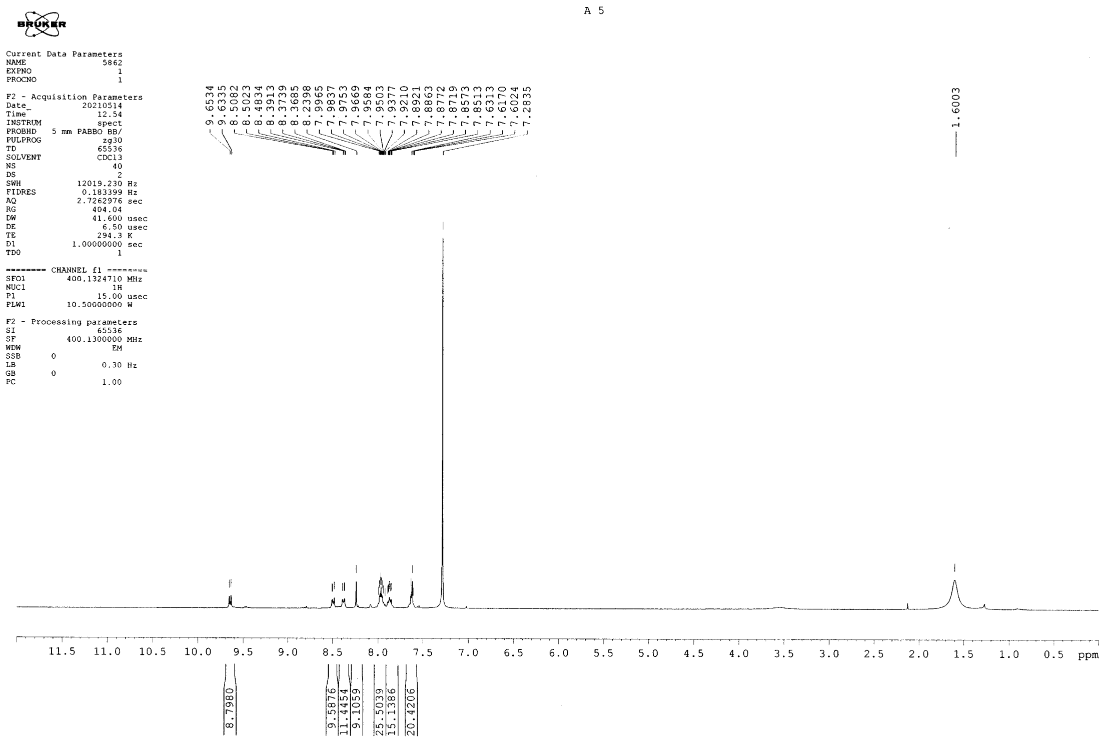

Enlarged spectrum in the range of 6-9 ppm.

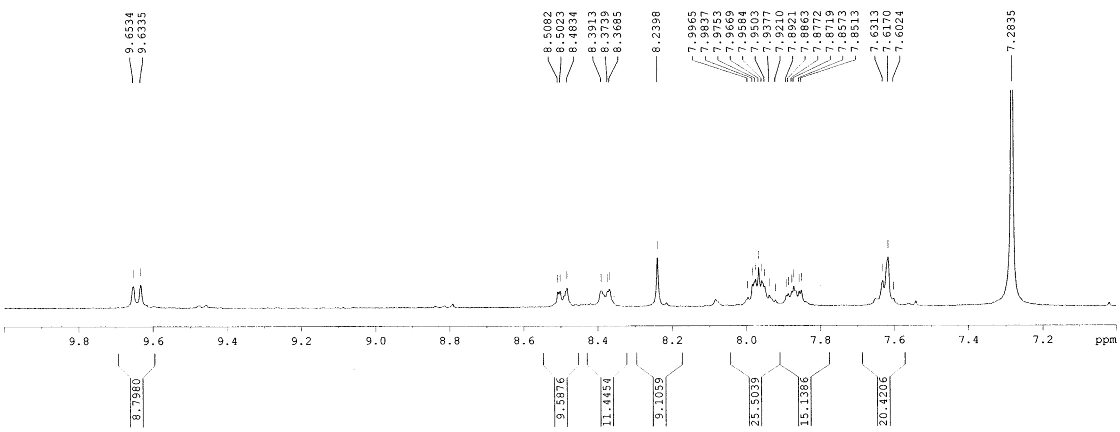

^{13}C spectrum of **FN6**

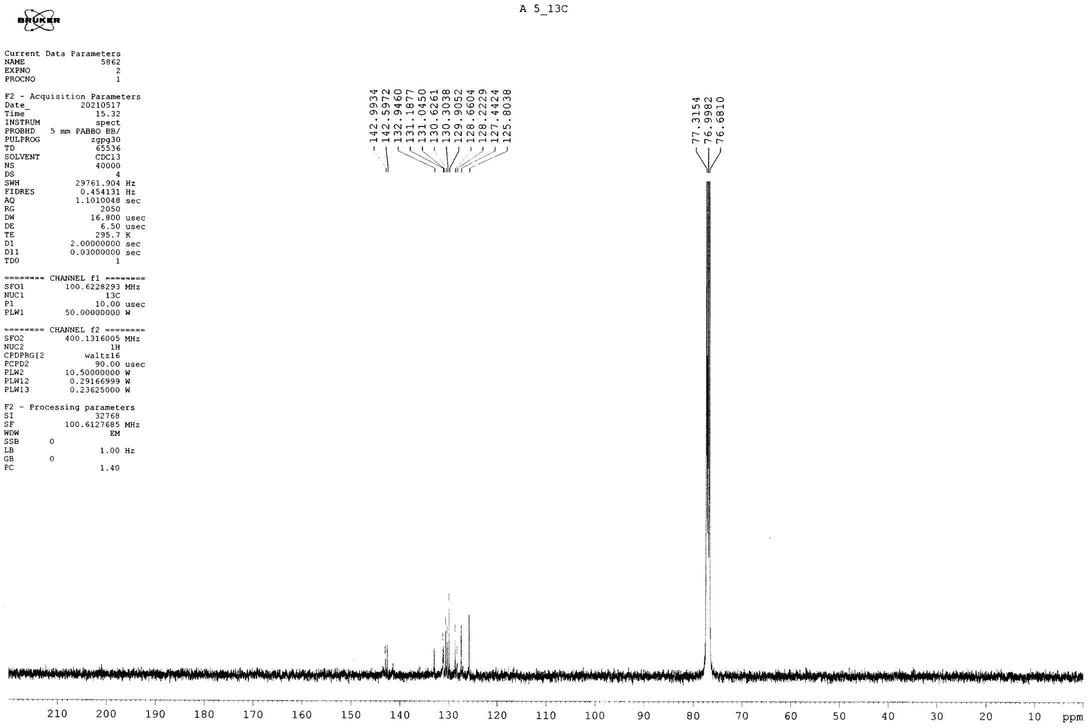

Enlarged spectrum in the range of 110-160 ppm.

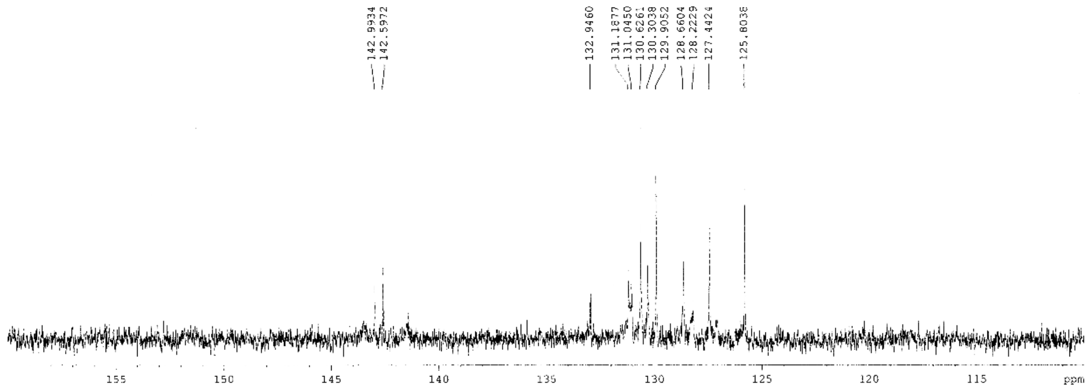

^{1}H spectrum of **FN7**

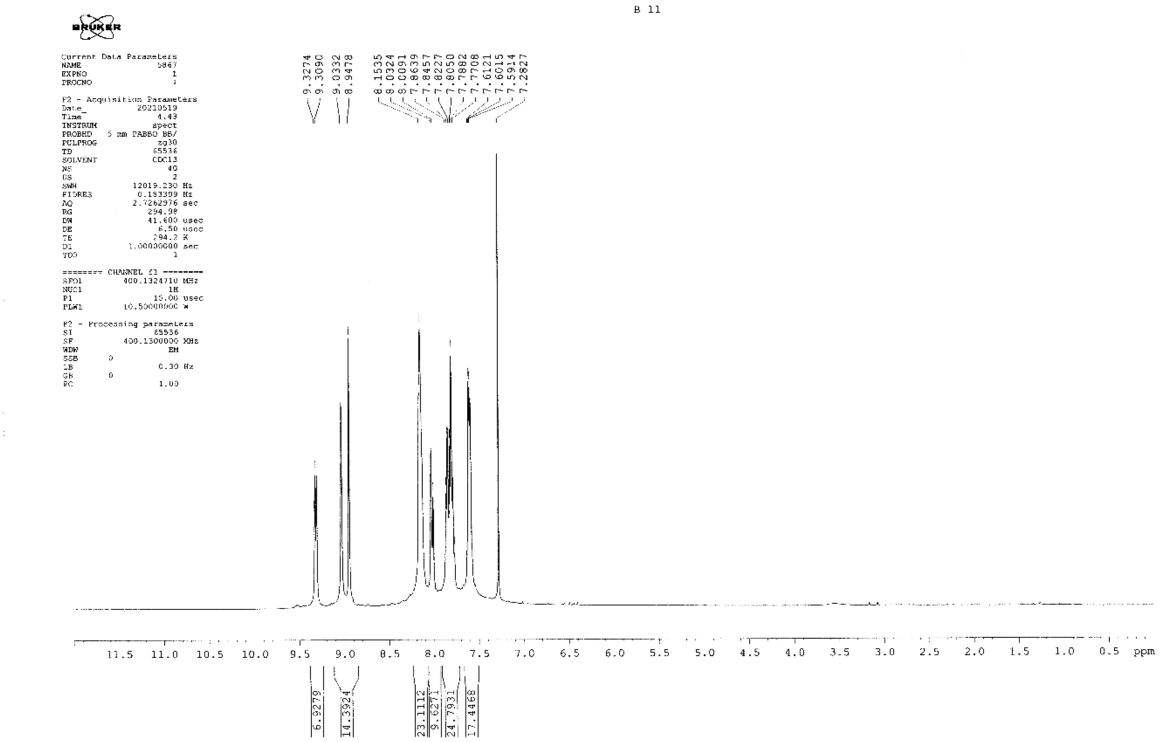

Enlarged spectrum in the range of 6-9 ppm.

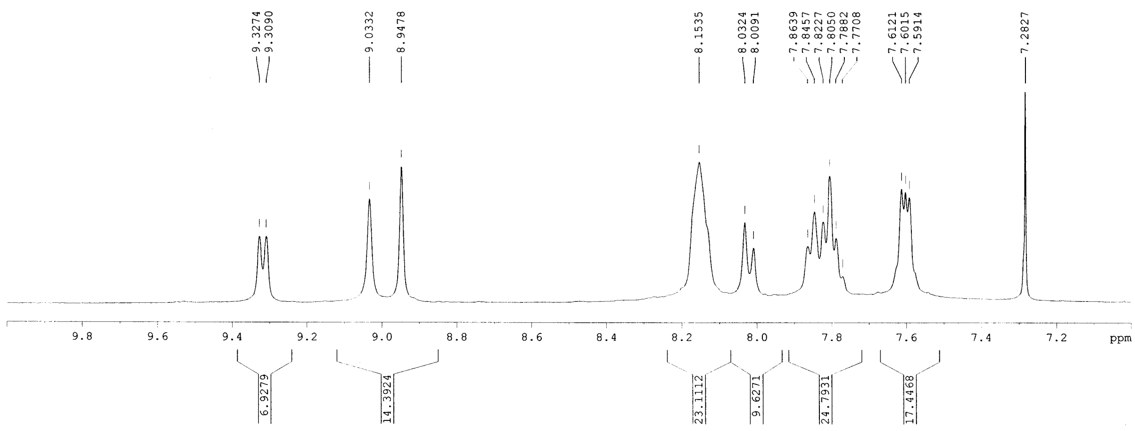

^{13}C spectrum of **FN7**

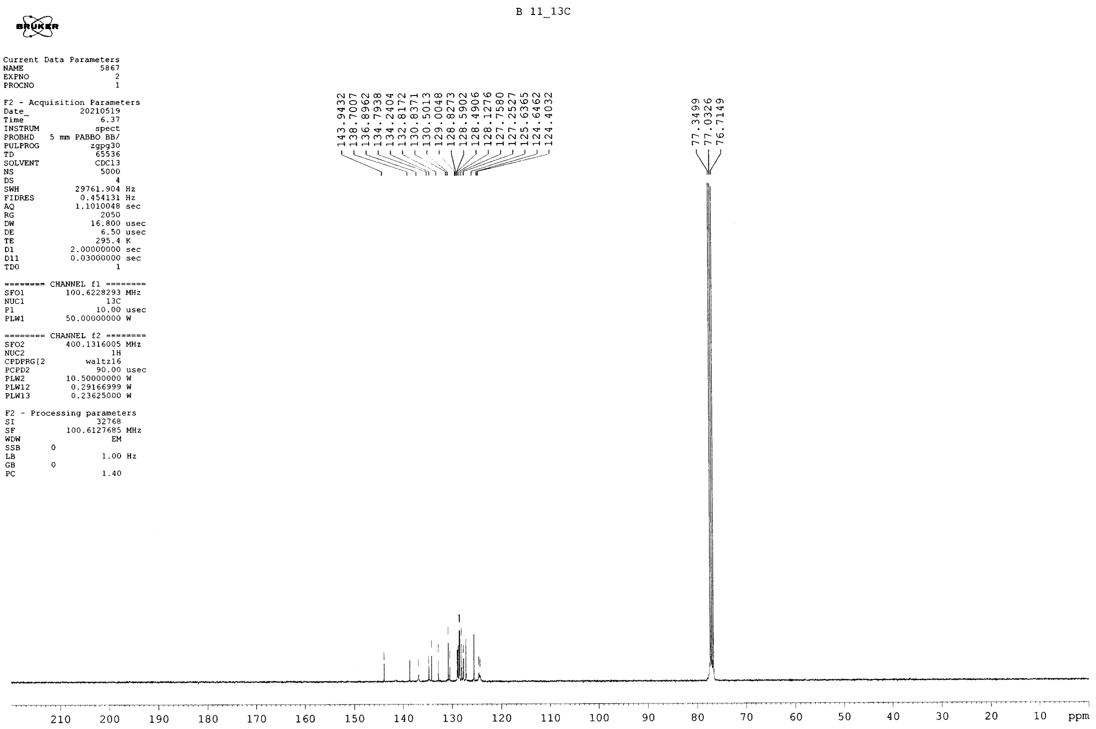

Enlarged spectrum in the range of 110-160 ppm.

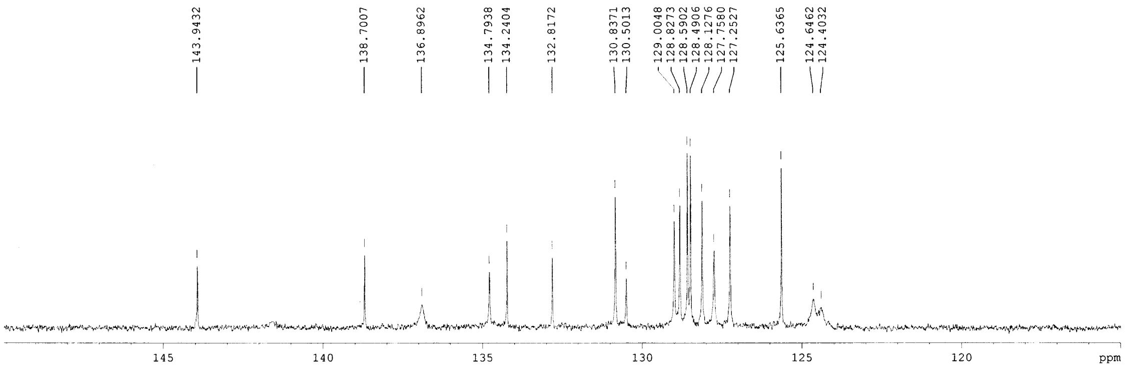

^{1}H spectrum of **FN8**

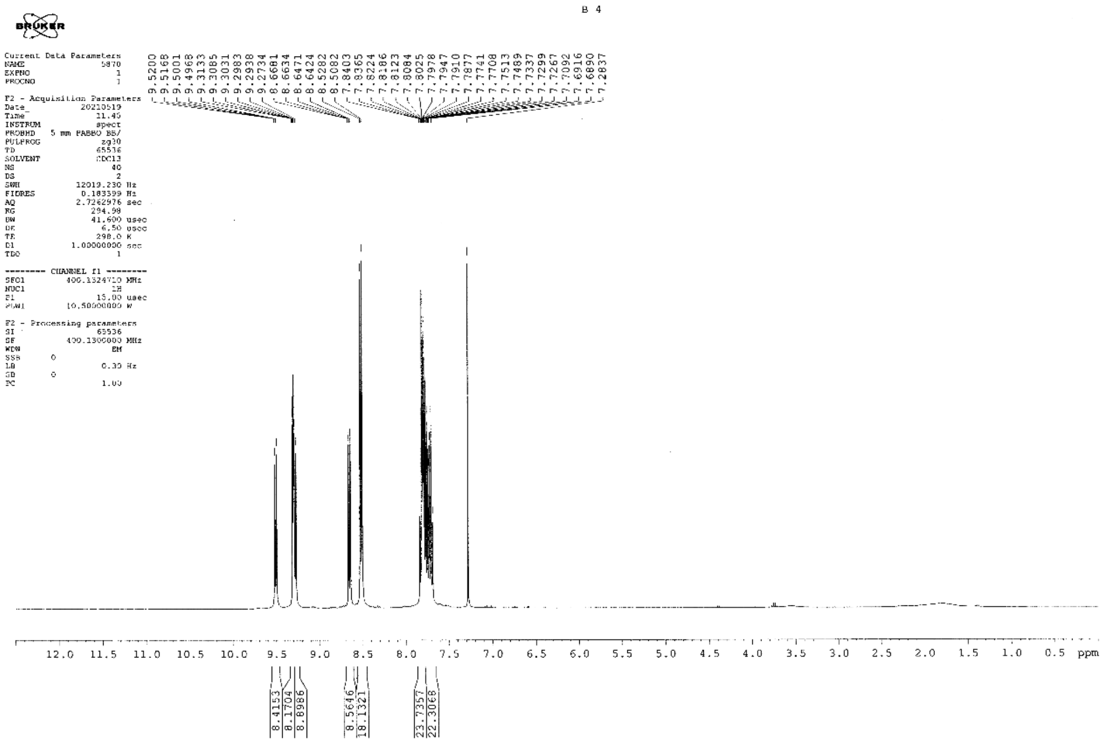

Enlarged spectrum in the range of 6-9 ppm.

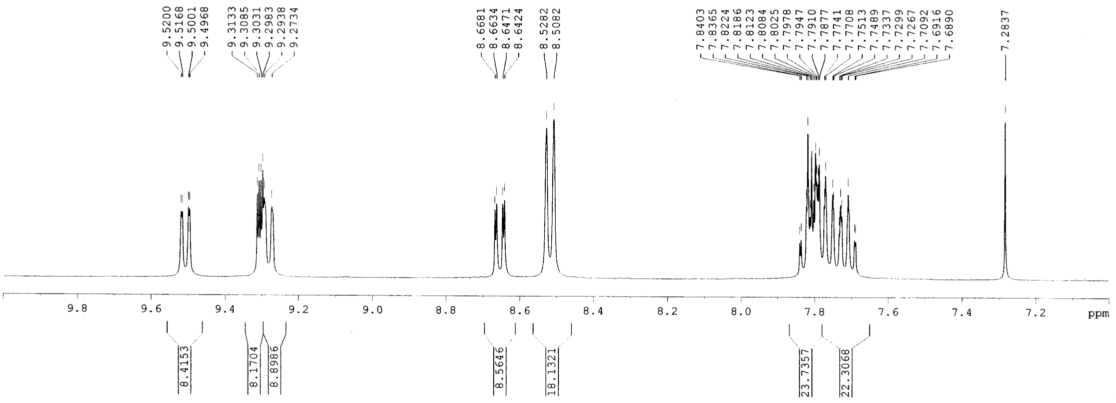

^{13}C spectrum of **FN8**

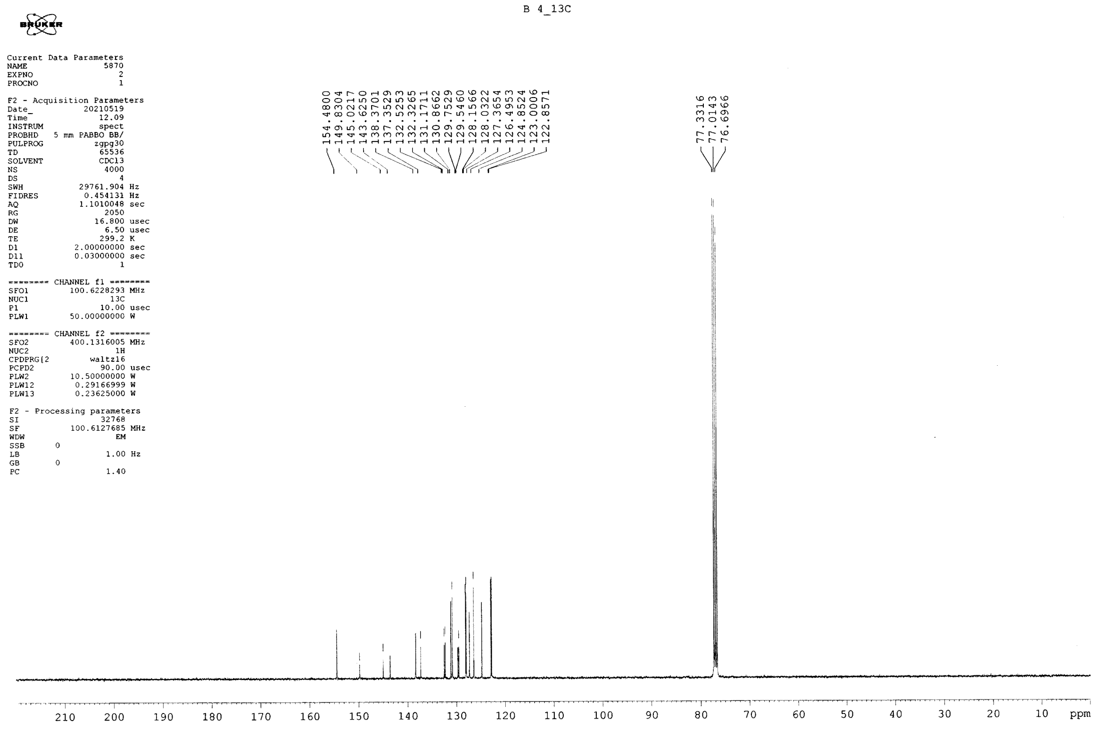

Enlarged spectrum in the range of 110-150 ppm.

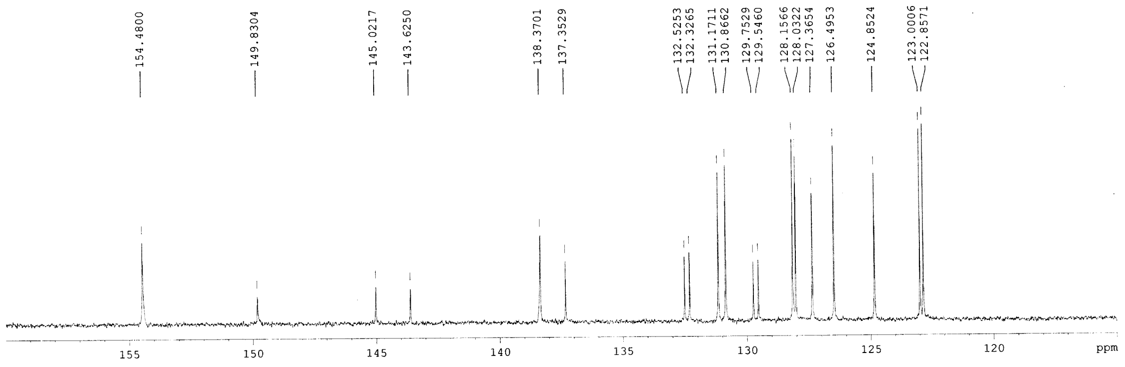

^{1}H spectrum of **FN9**

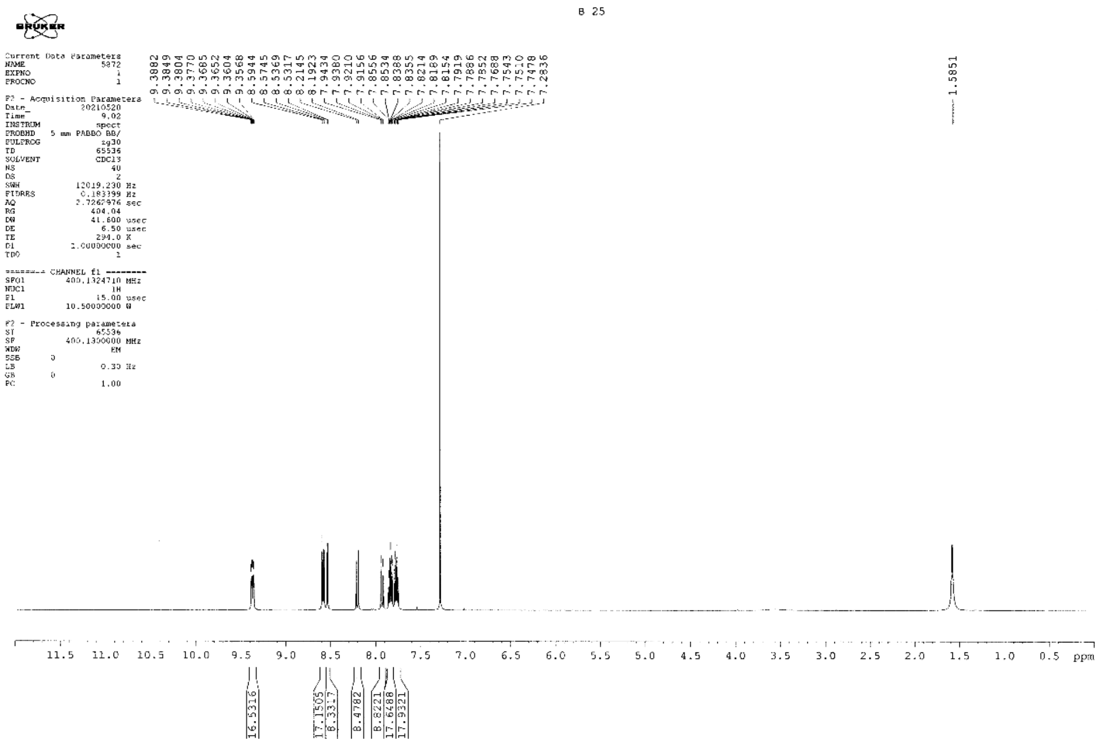

Enlarged spectrum in the range of 6-9 ppm.

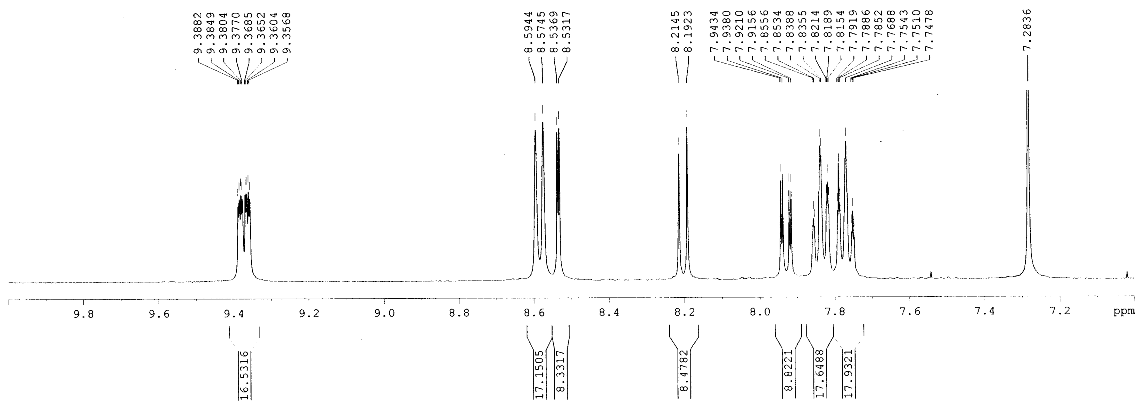

^{13}C spectrum of **FN9**

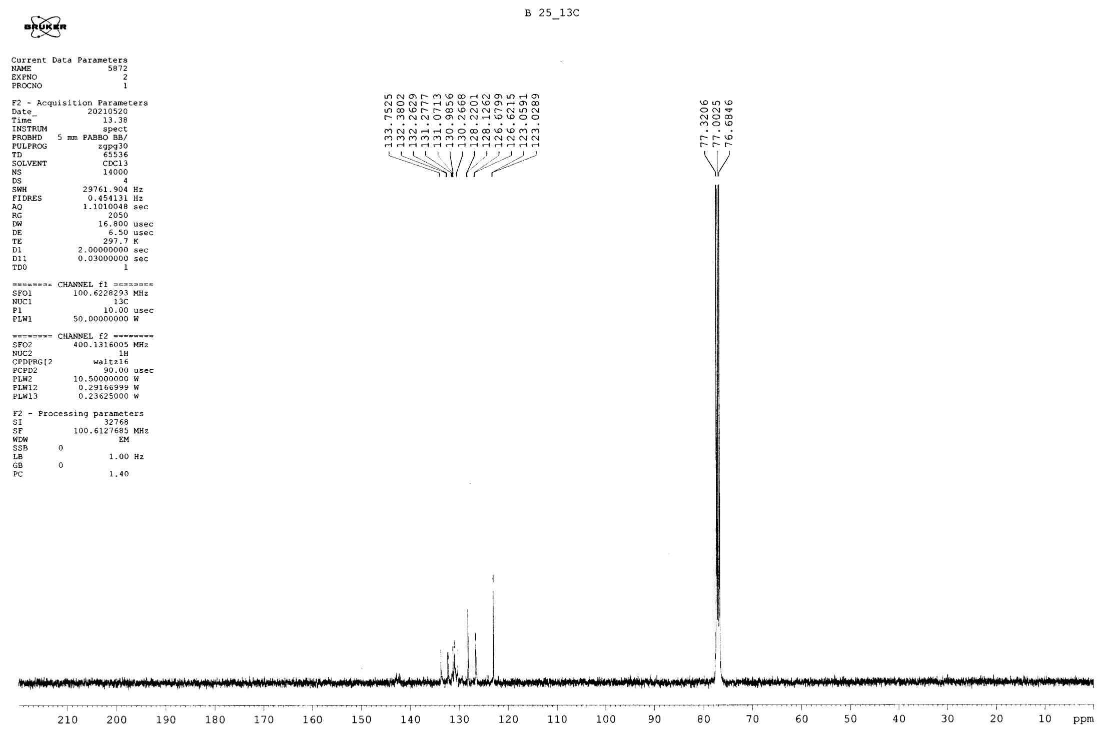

Enlarged spectrum in the range of 110-150 ppm.

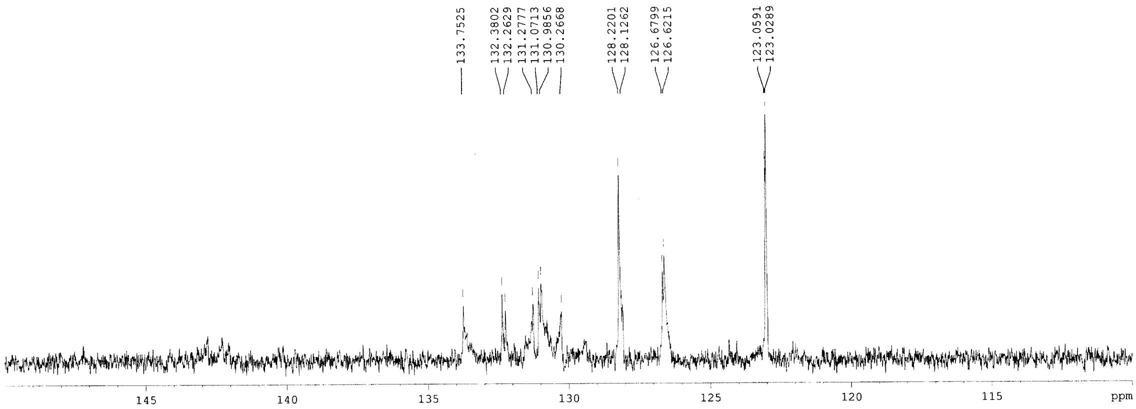

^1H spectrum of **FN10**

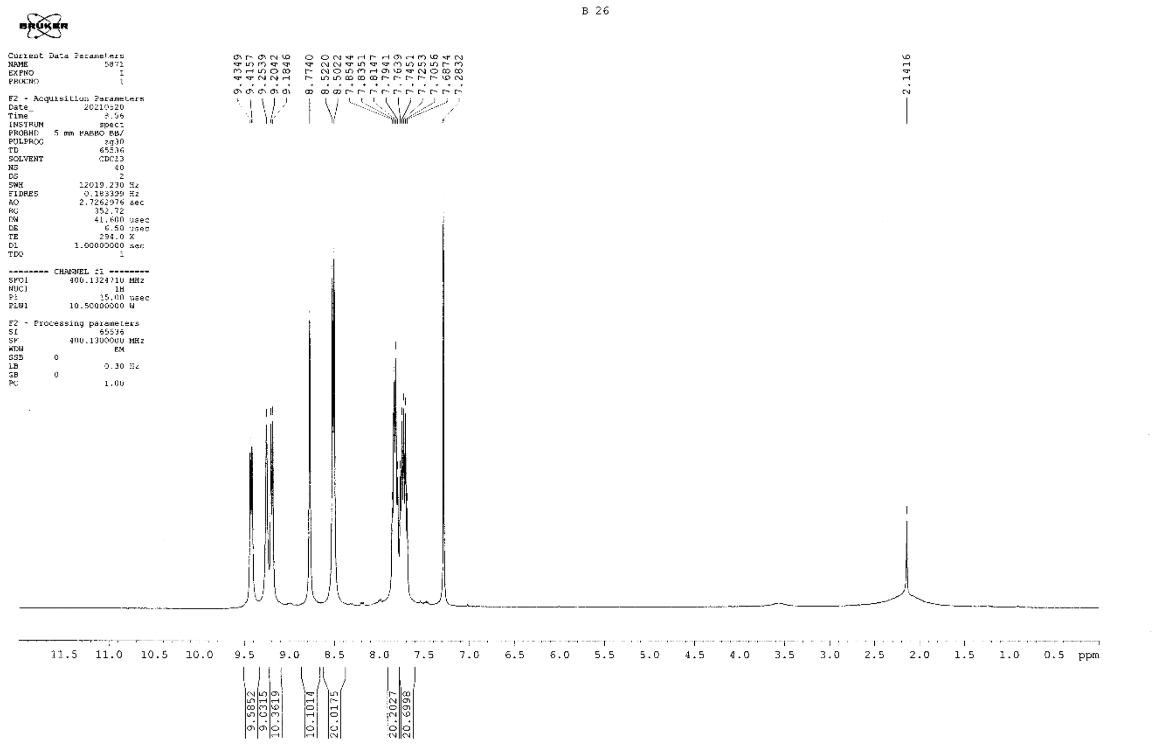

Enlarged spectrum in the range of 6-9 ppm.

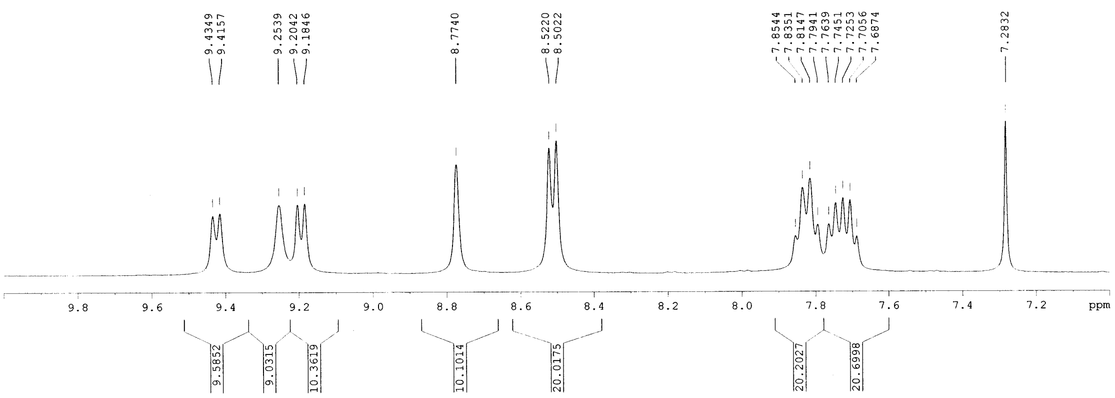

^{13}C spectrum of **FN10**

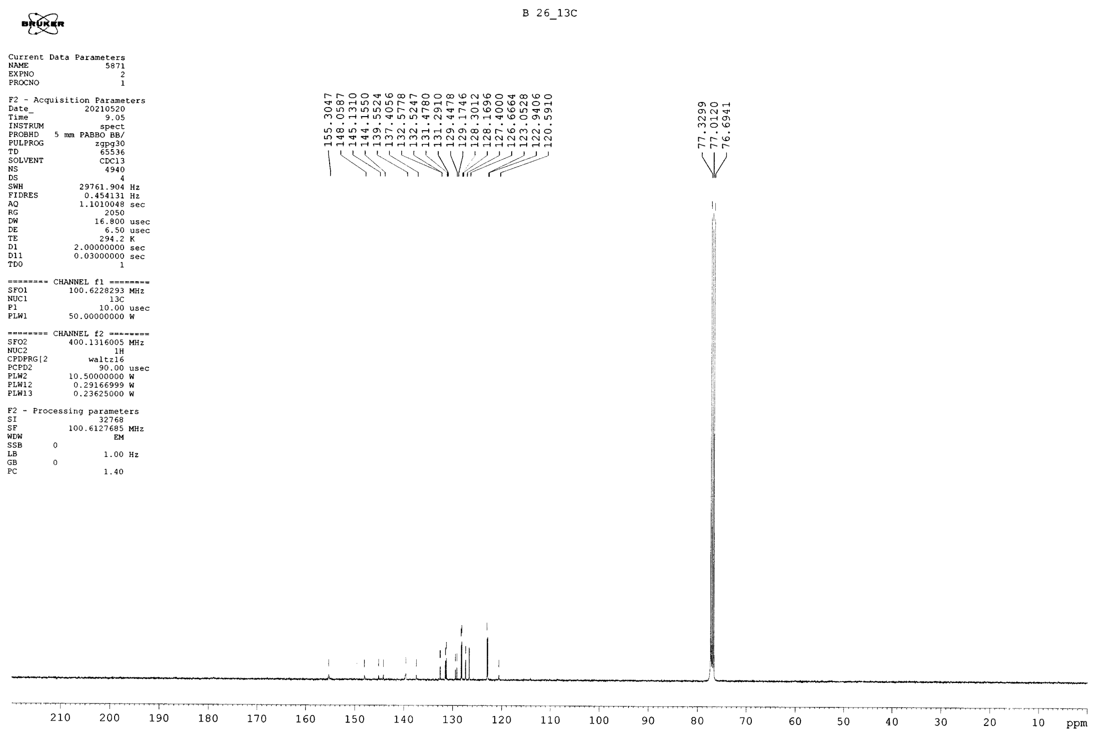

Enlarged spectrum in the range of 110-150 ppm.

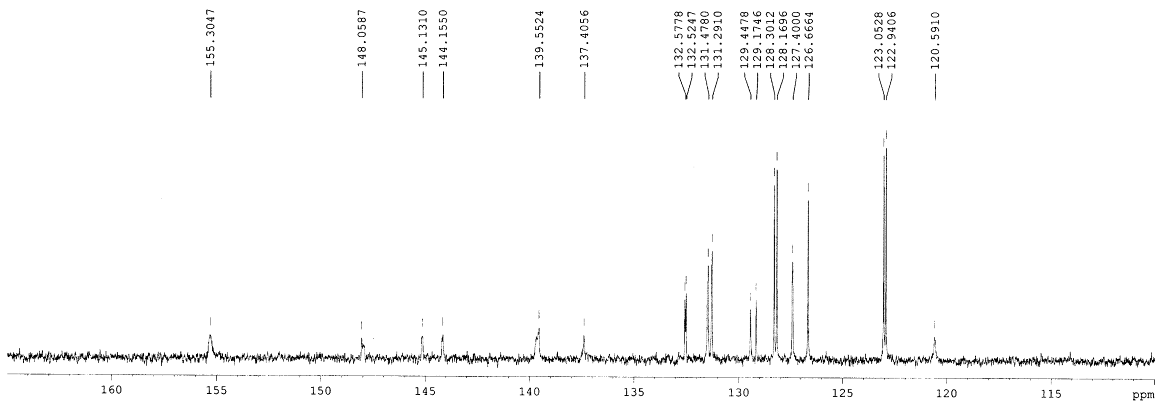

^{1}H spectrum of **FN11**

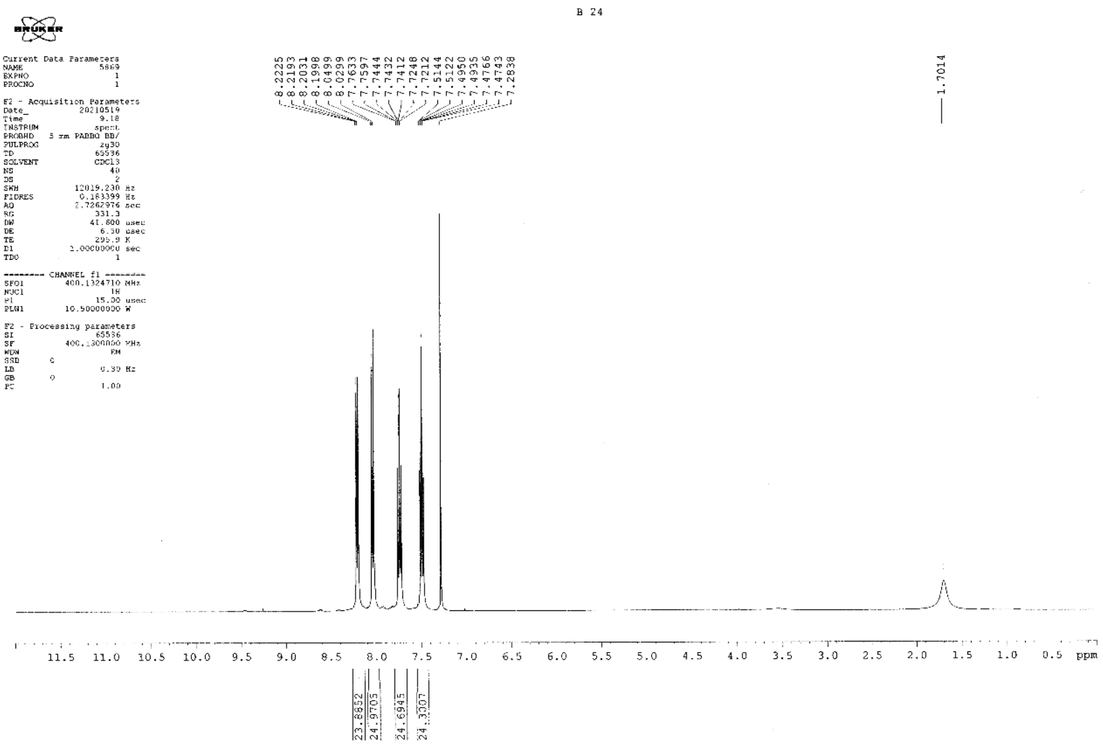

Enlarged spectrum in the range of 6-9 ppm.

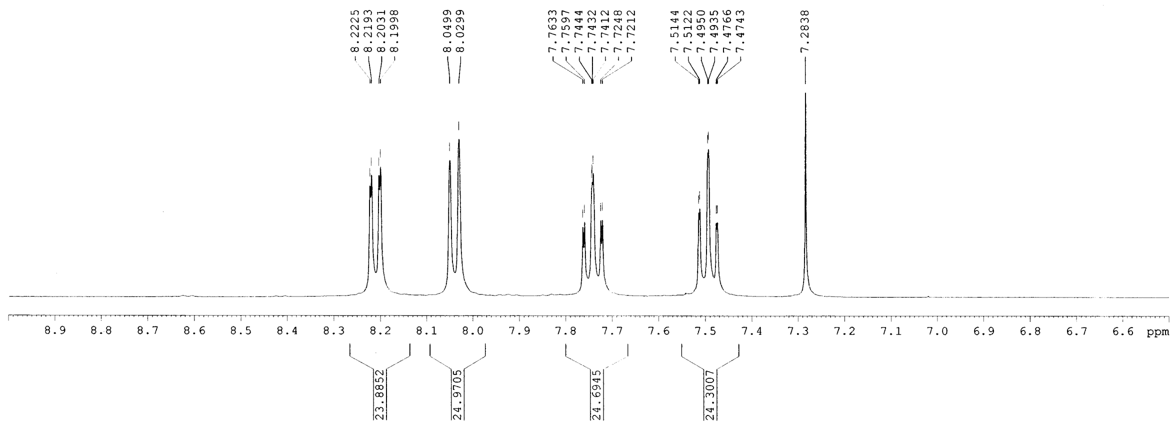

^{13}C spectrum of **FN11**

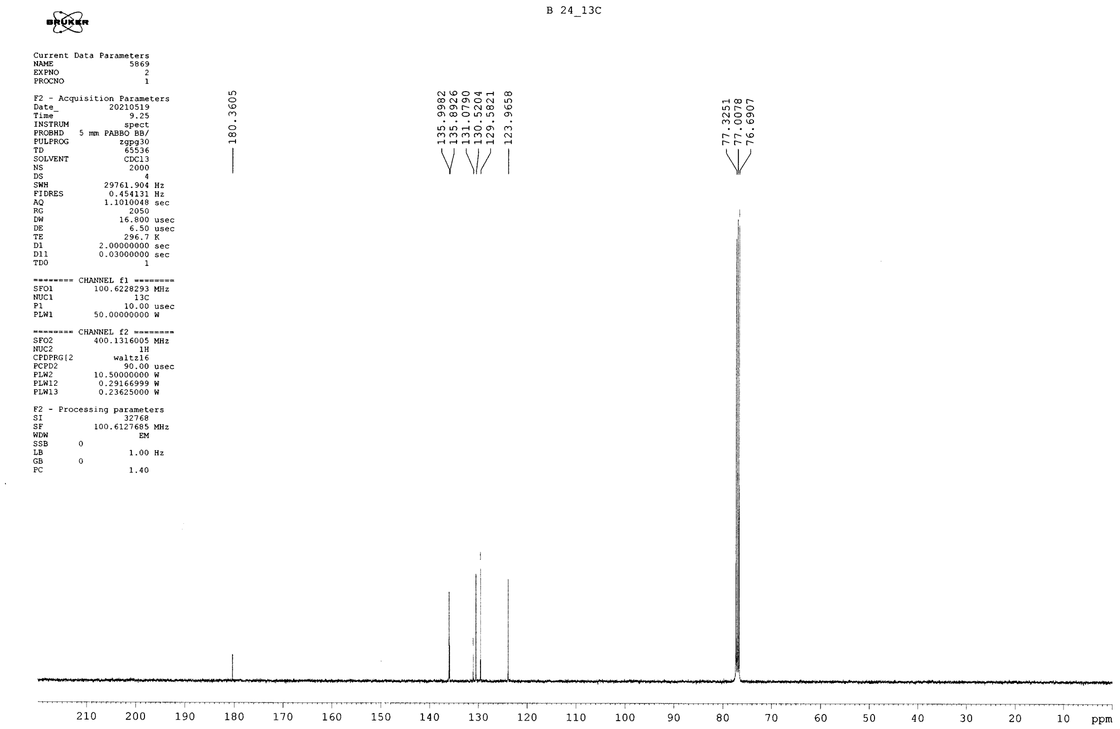

Enlarged spectrum in the range of 110-150 ppm.

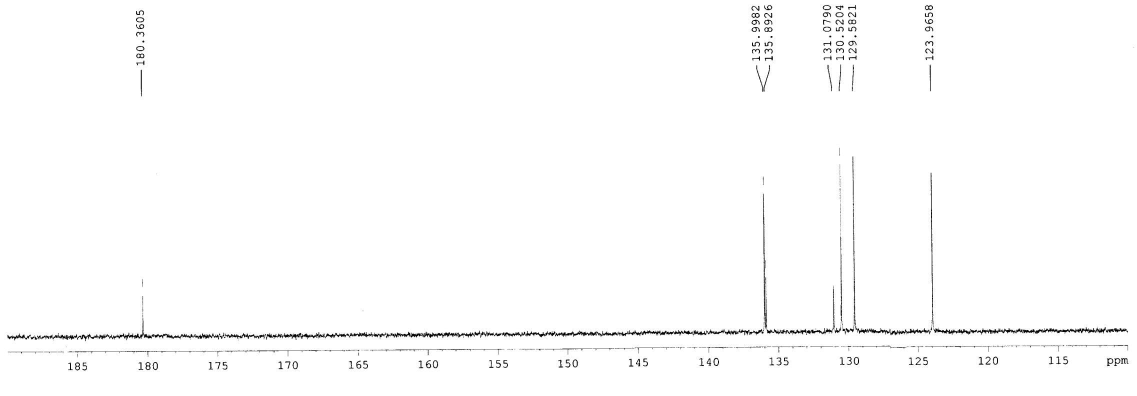

^{1}H spectrum of **FN12**

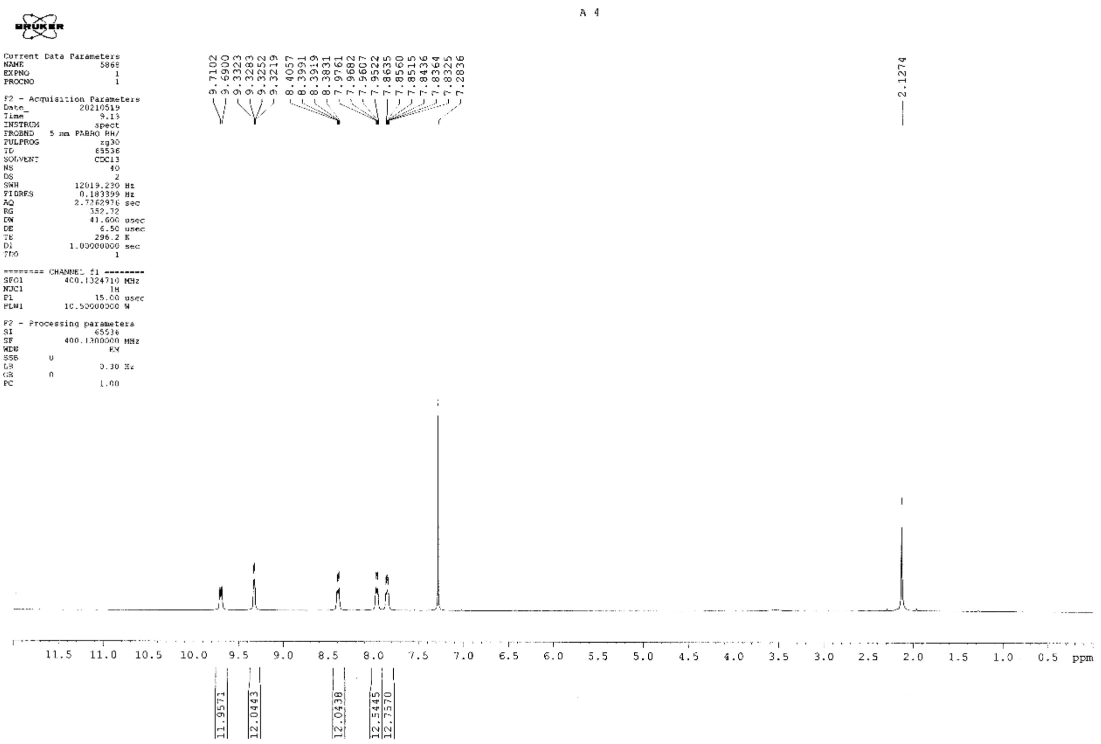

Enlarged spectrum in the range of 6-9 ppm.

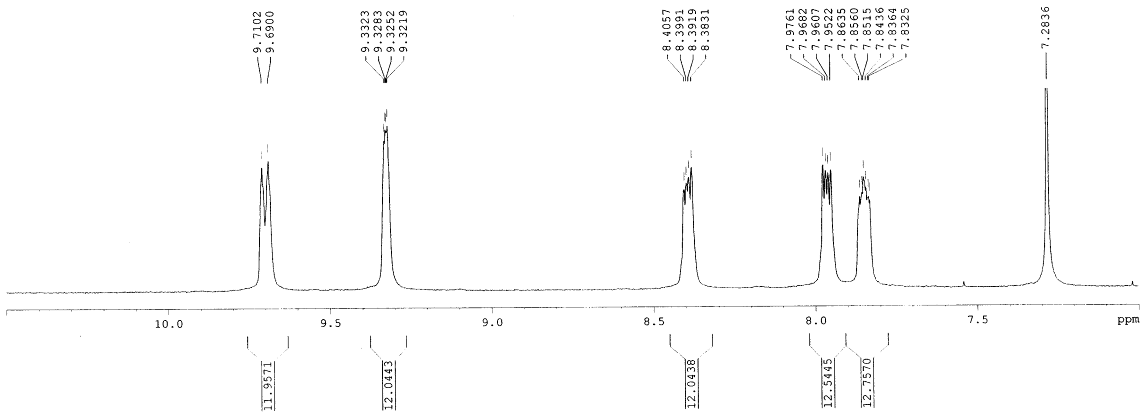

^{13}C spectrum of **FN12**

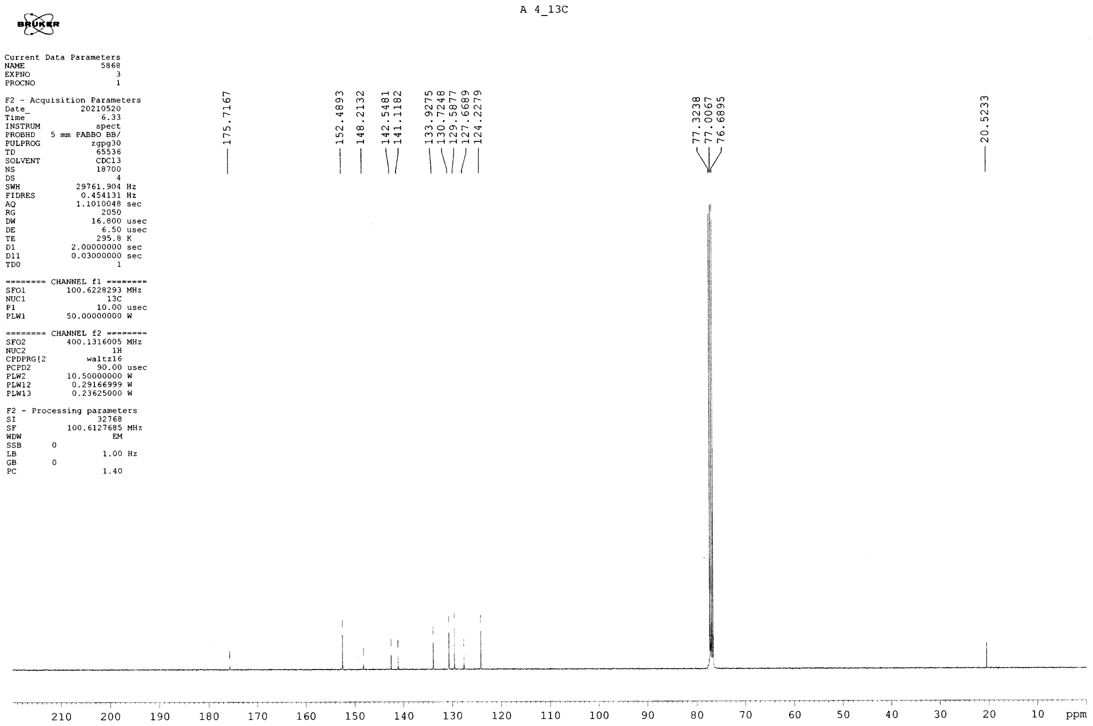

Enlarged spectrum in the range of 100-150 ppm.

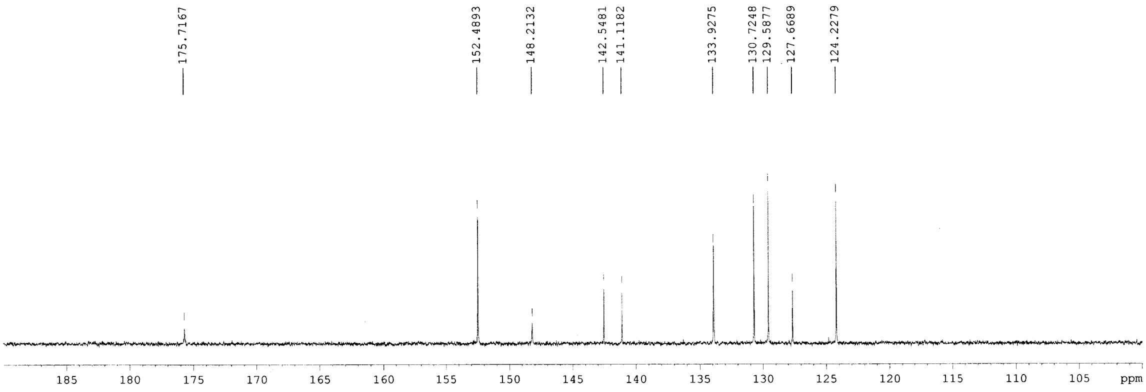

^{1}H spectrum of **FN13**

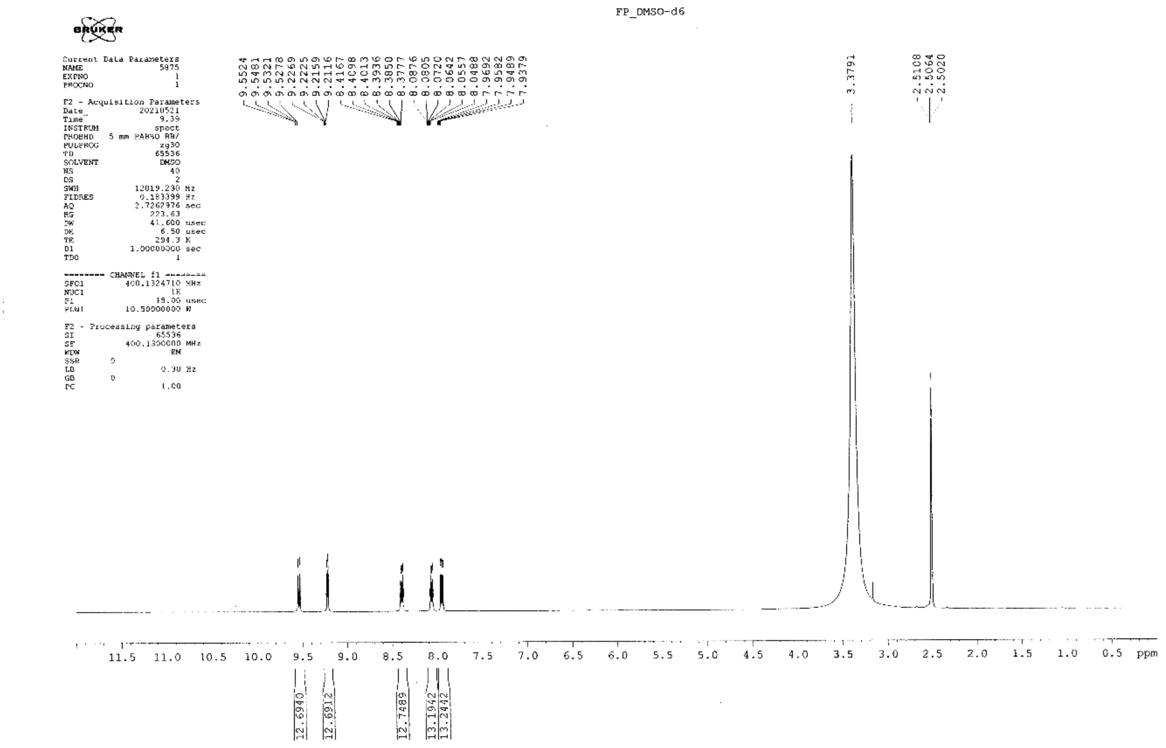

Enlarged spectrum in the range of 100-150 ppm.

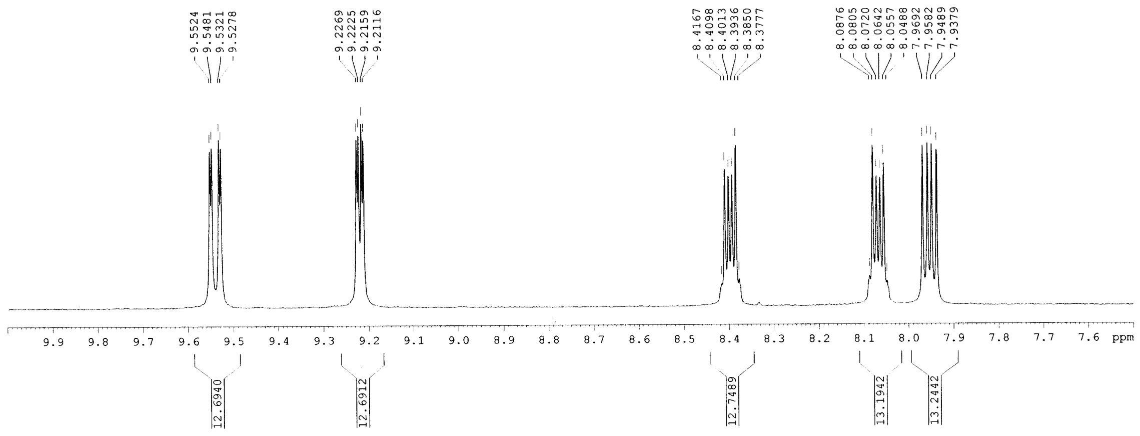

^{13}C spectrum of **FN13**

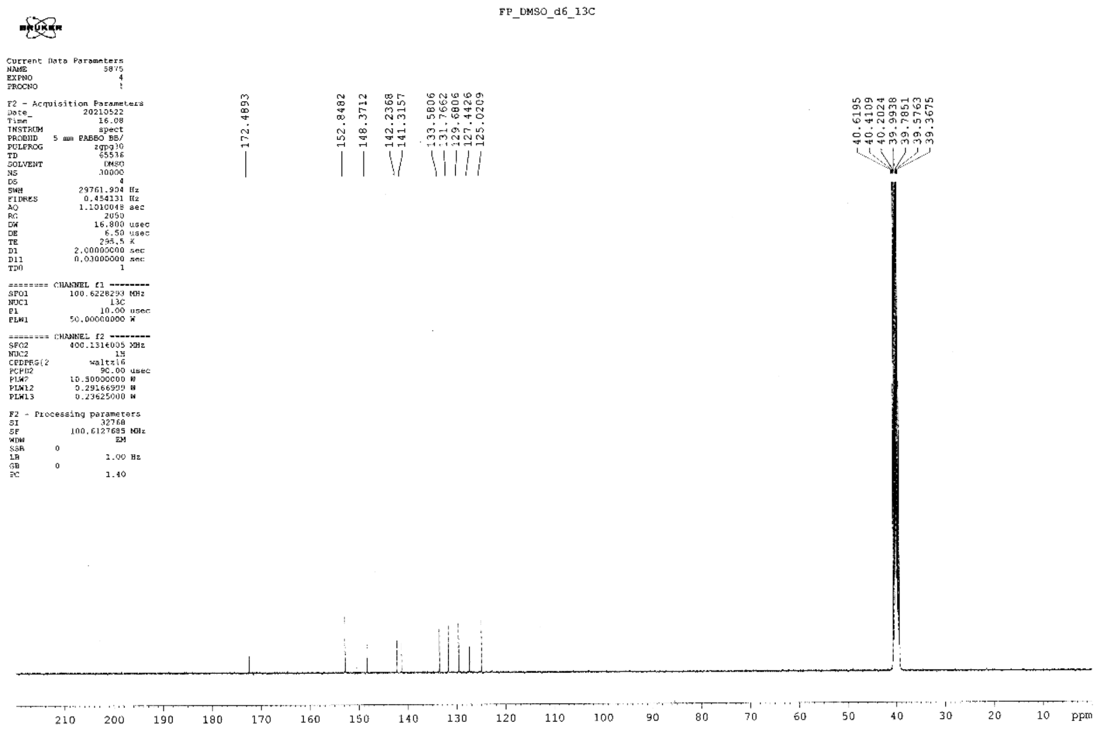

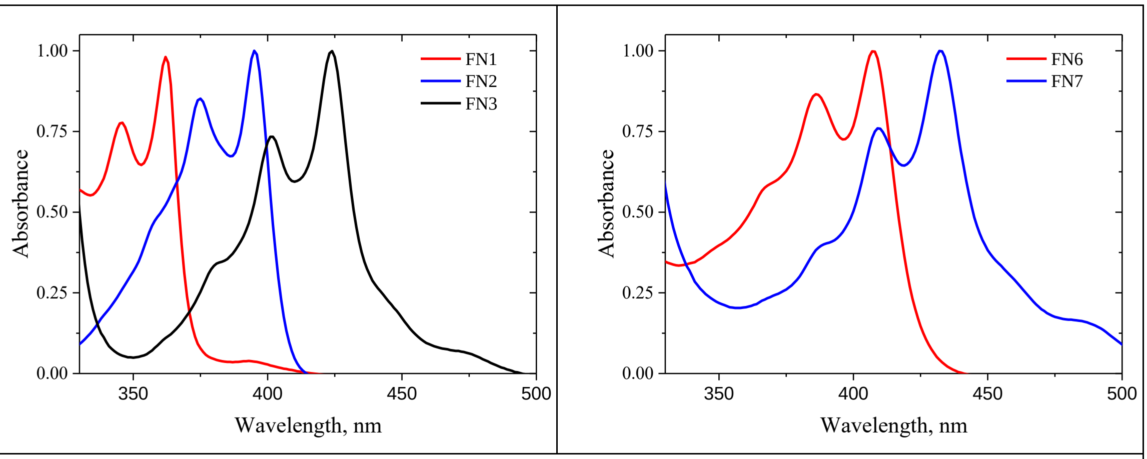

(**A**) the number of conjugated double bonds in the molecule

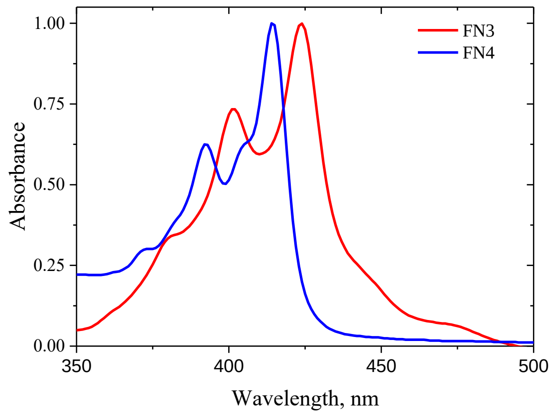

(**B**) angular arrangement of the rings

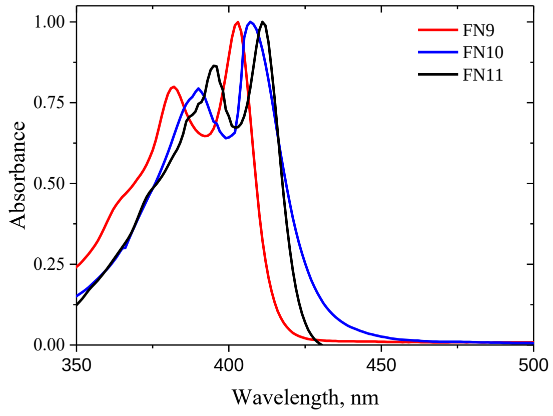

(**C**) increase in the number of nitrogen atoms in the molecule

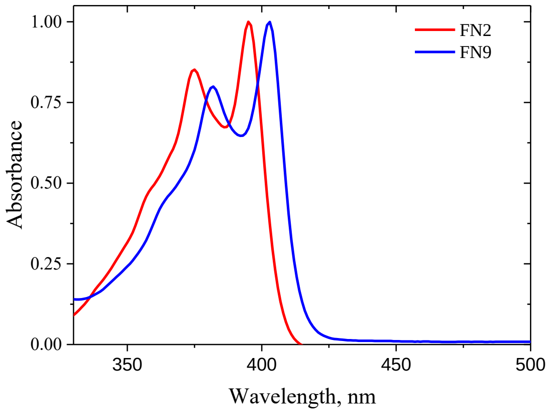

(**D**) the presence of a heavy atom

Figure S1. Normalized electronic absorption spectra of phenazine derivatives in ethyl acetate illustrating the influence of dye structure on the bands position.

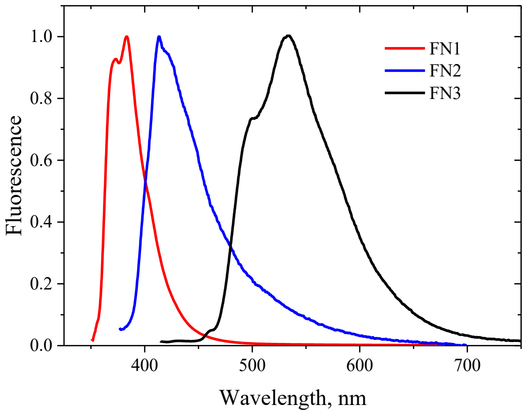

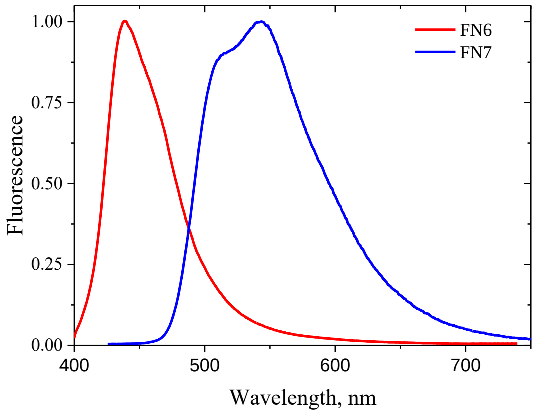

(**A**) the number of conjugated double bonds in the molecule

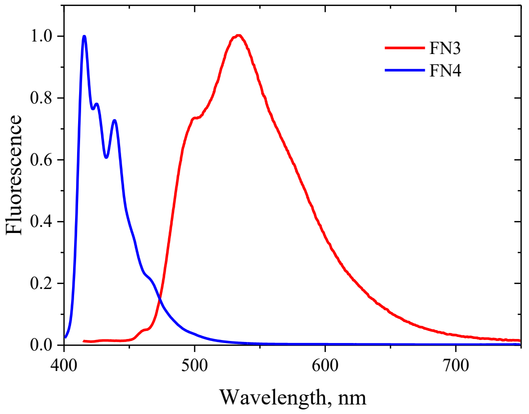

(**B**) angular arrangement of the rings

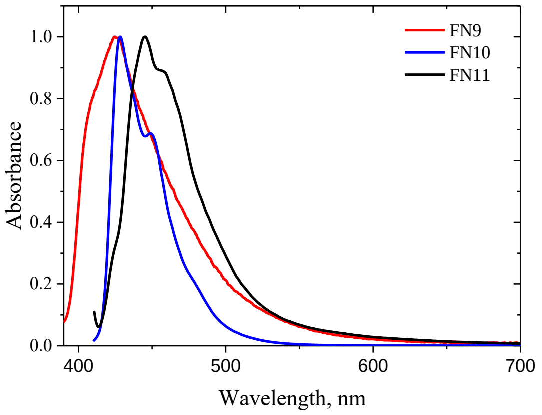

(**C**) increase in the number of nitrogen atoms in the molecule

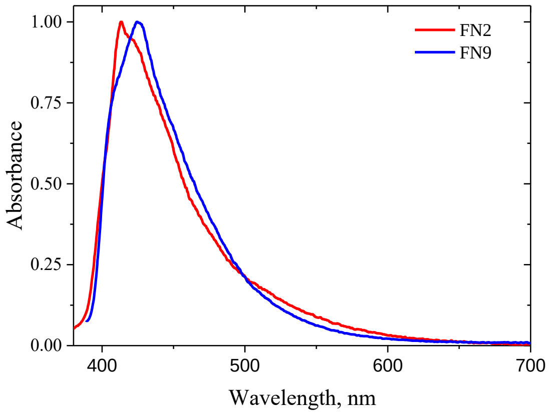

(**D**) the presence of a heavy atom

Figure S2. Normalized fluorescence spectra of phenazine derivatives in ethyl acetate illustrating the influence of dye structure on the bands position; Ex = 366 nm or 404 nm.

^{1}H NMR spectra of a mixture of dibenzo[a.c]phenazine **FN2** (sensitizer) and 2,3-diphenyl-*p*-dioxene in $CDCl_3$ after different irradiation time

^{1}H NMR spectrum before irradiation.

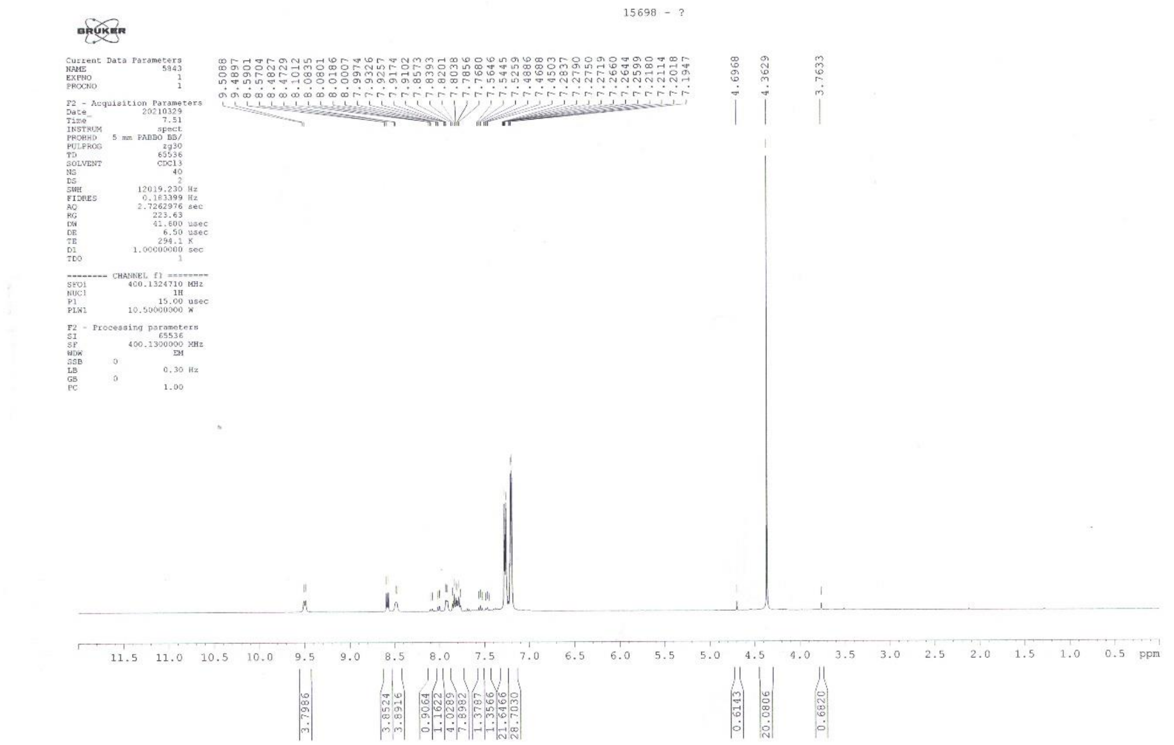

^{1}H NMR spectrum after 10 min. irradiation.

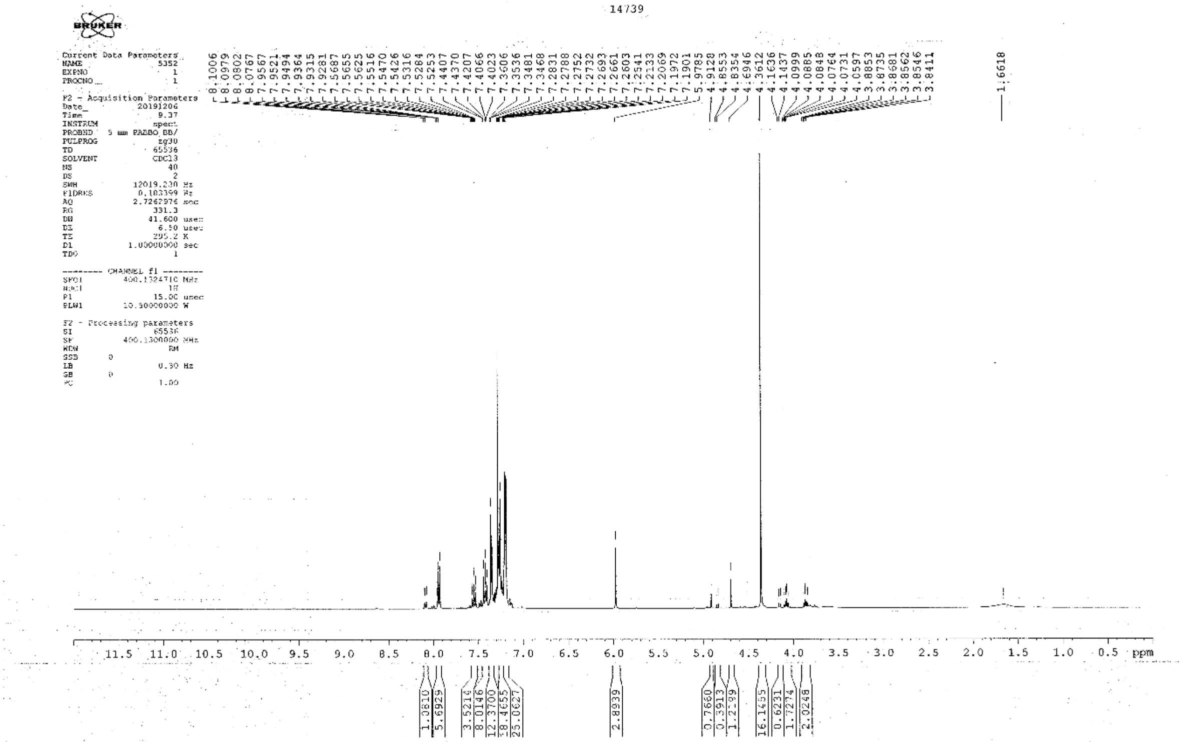

Enlarged spectrum in the range of 0-4 ppm and 3-5 ppm after 10 min. irradiation.

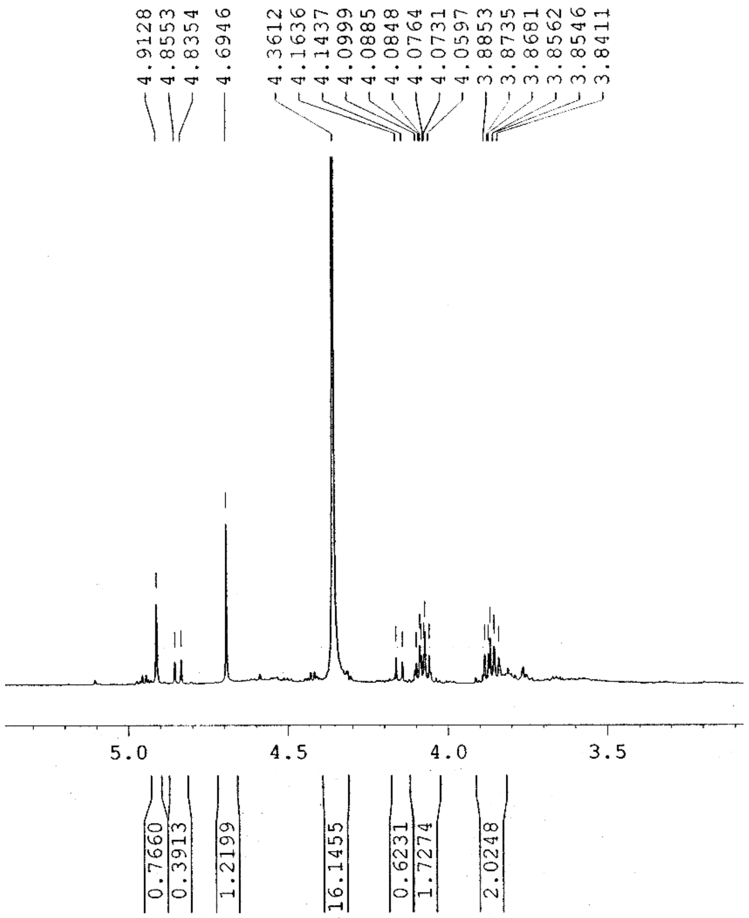

^{1}H NMR spectrum after 20 min. irradiation.

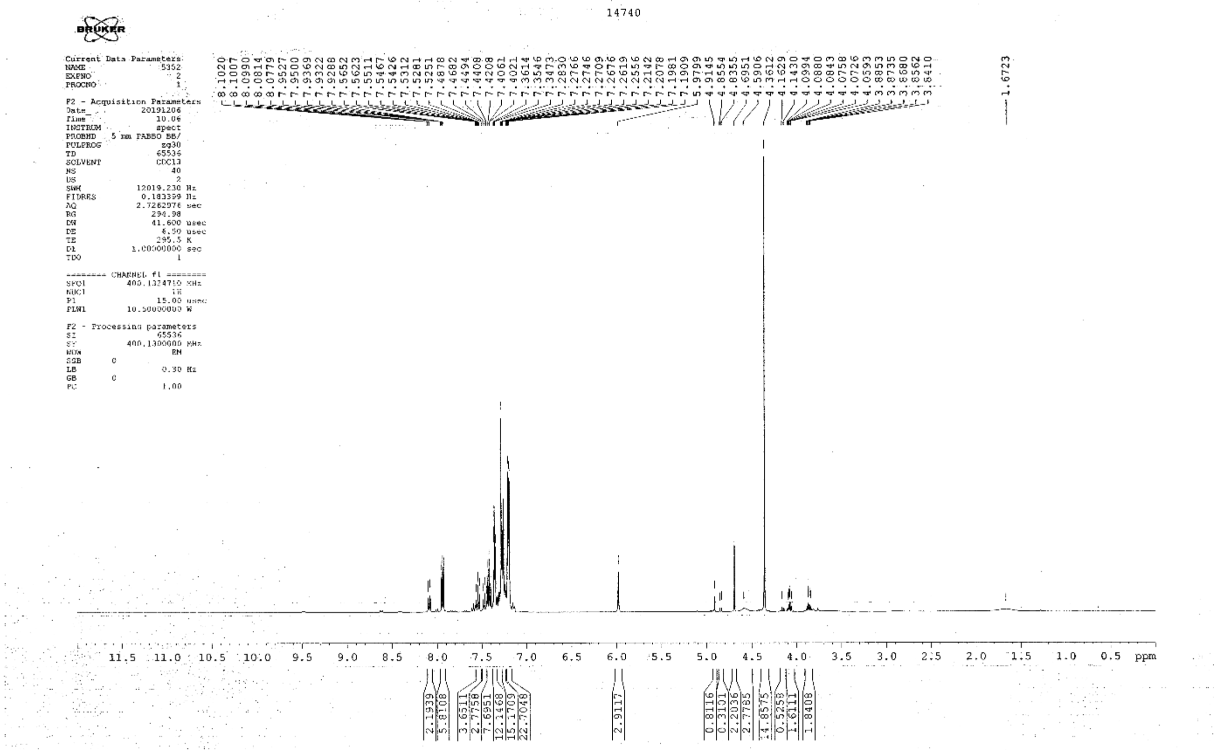

Enlarged spectrum in the range of 0-4 ppm and 3-5 ppm after 20 min. irradiation.

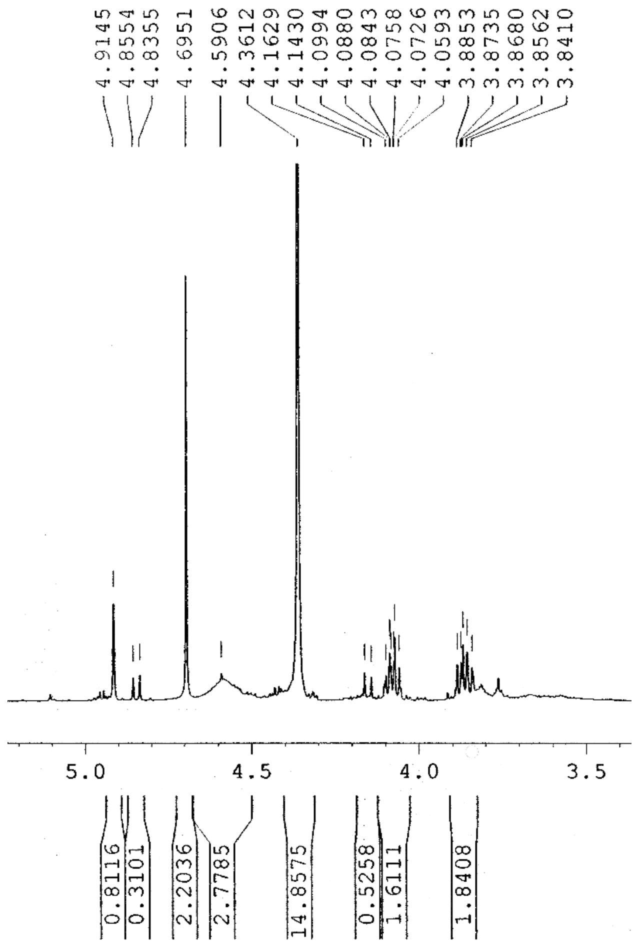

^{1}H NMR spectrum after 30 min. irradiation.

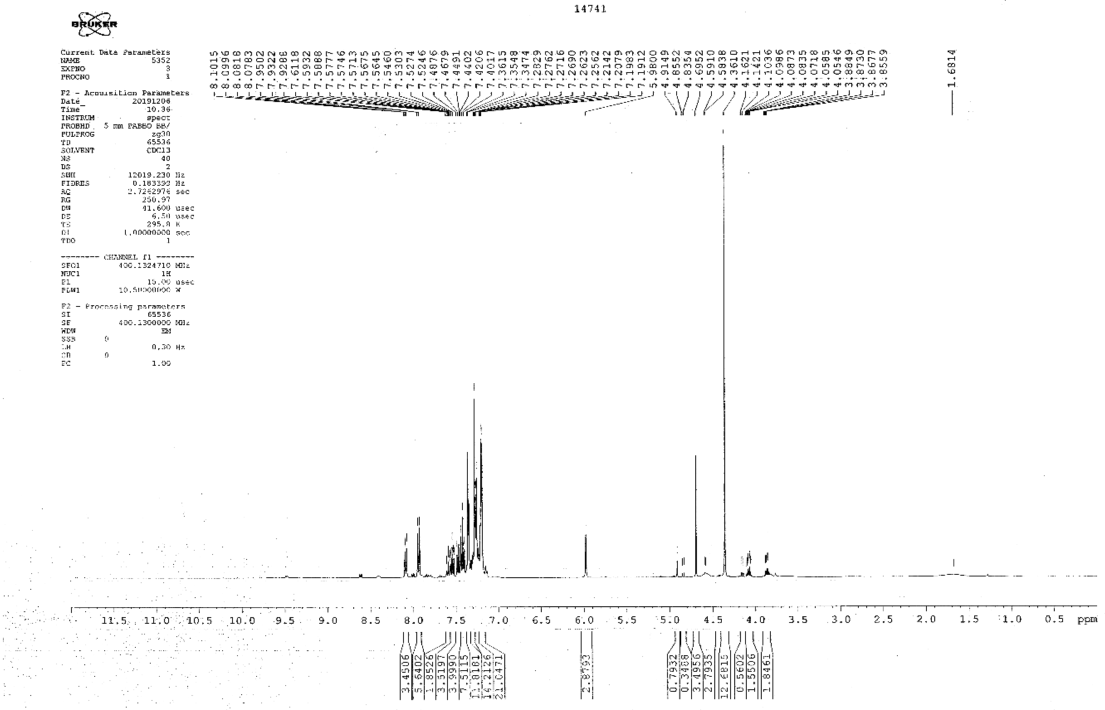

Enlarged spectrum in the range of 0-4 ppm and 3-5 ppm after 30 min. irradiation.

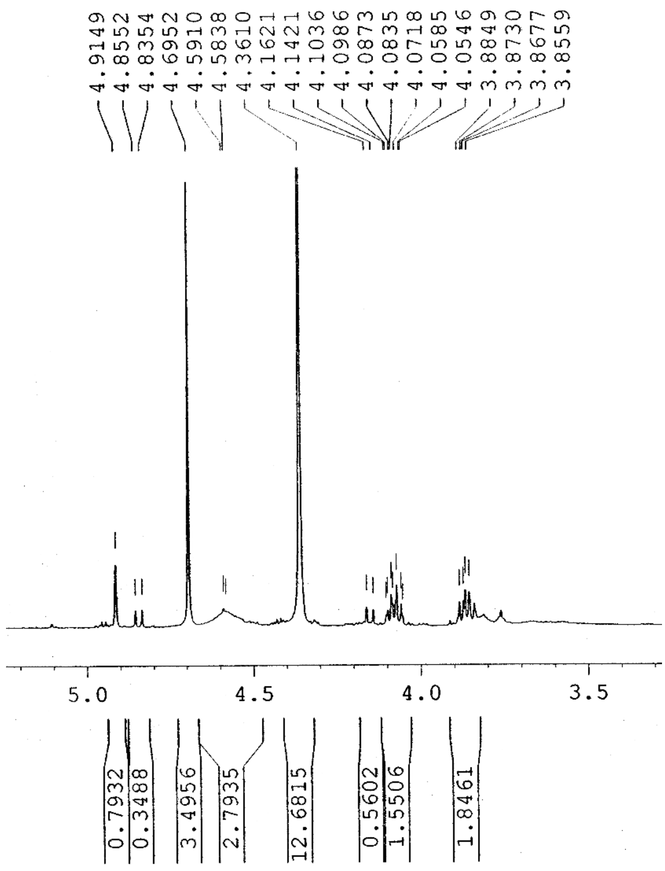

Figure S3. ^{1}H NMR spectra of a mixture of dibenzo[a.c]phenazine **FN2** (sensitizer) and 2,3-diphenyl-*p*-dioxene in $CDCl_3$ after different irradiation time; dental lamp, 18 mW.
